# Supplementary figures and images for: Delilah, prospero, and D-Pax2 constitute a gene regulatory network essential for the development of functional proprioceptors
Source: eLife. 2021 Dec 29;10:e70833. doi: 10.7554/eLife.70833 (PMC8716109; doi:10.7554/eLife.70833)

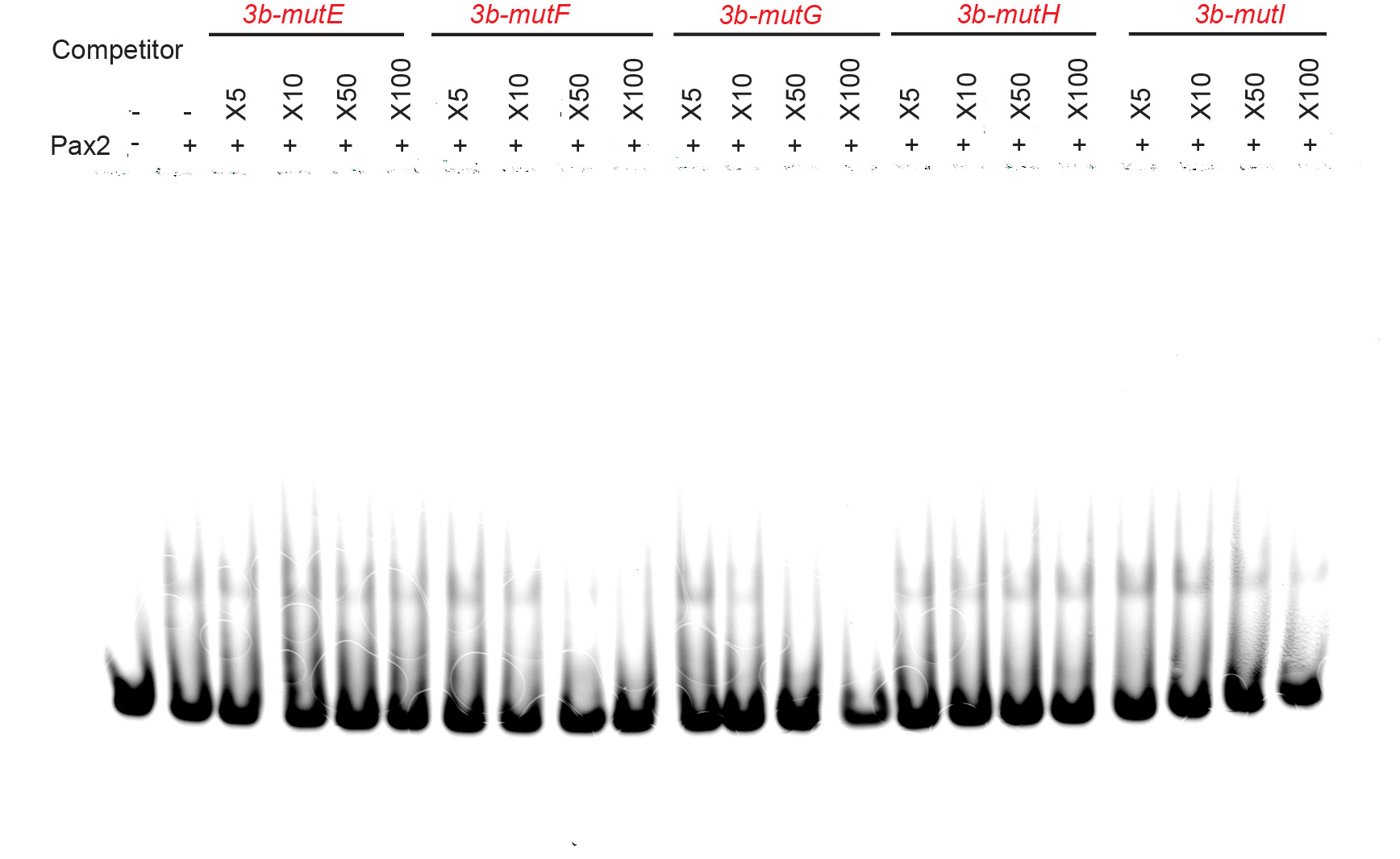

Supplement: Figure 5—figure supplement 1—source data 1. [file elife-70833-fig5-figsupp1-data1.zip › Figure5 - figure supplement 1 - source data 1/Figure 5-S1D - Source data 2_labeled.jpg]

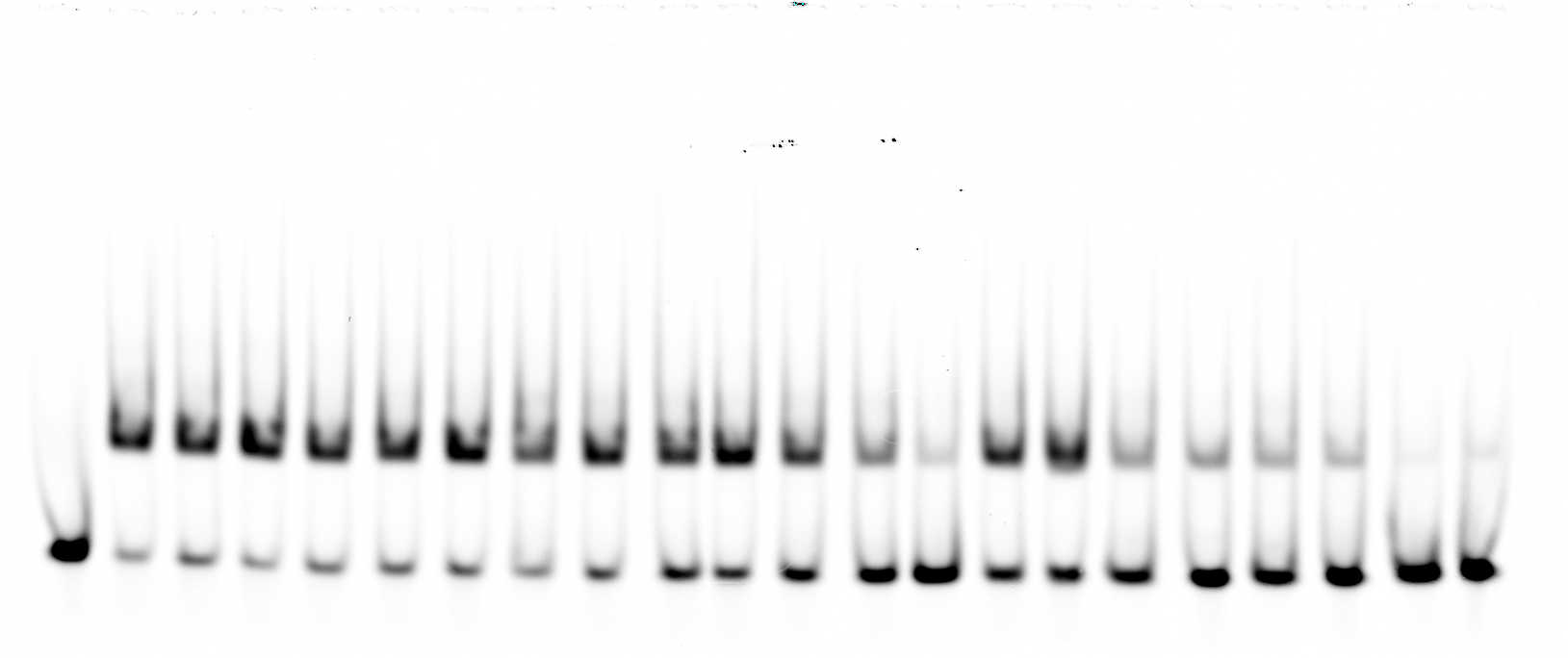

Supplement: Figure 5—figure supplement 1—source data 1. [file elife-70833-fig5-figsupp1-data1.zip › Figure5 - figure supplement 1 - source data 1/Figure 5-S15E - Source data 2.tif]

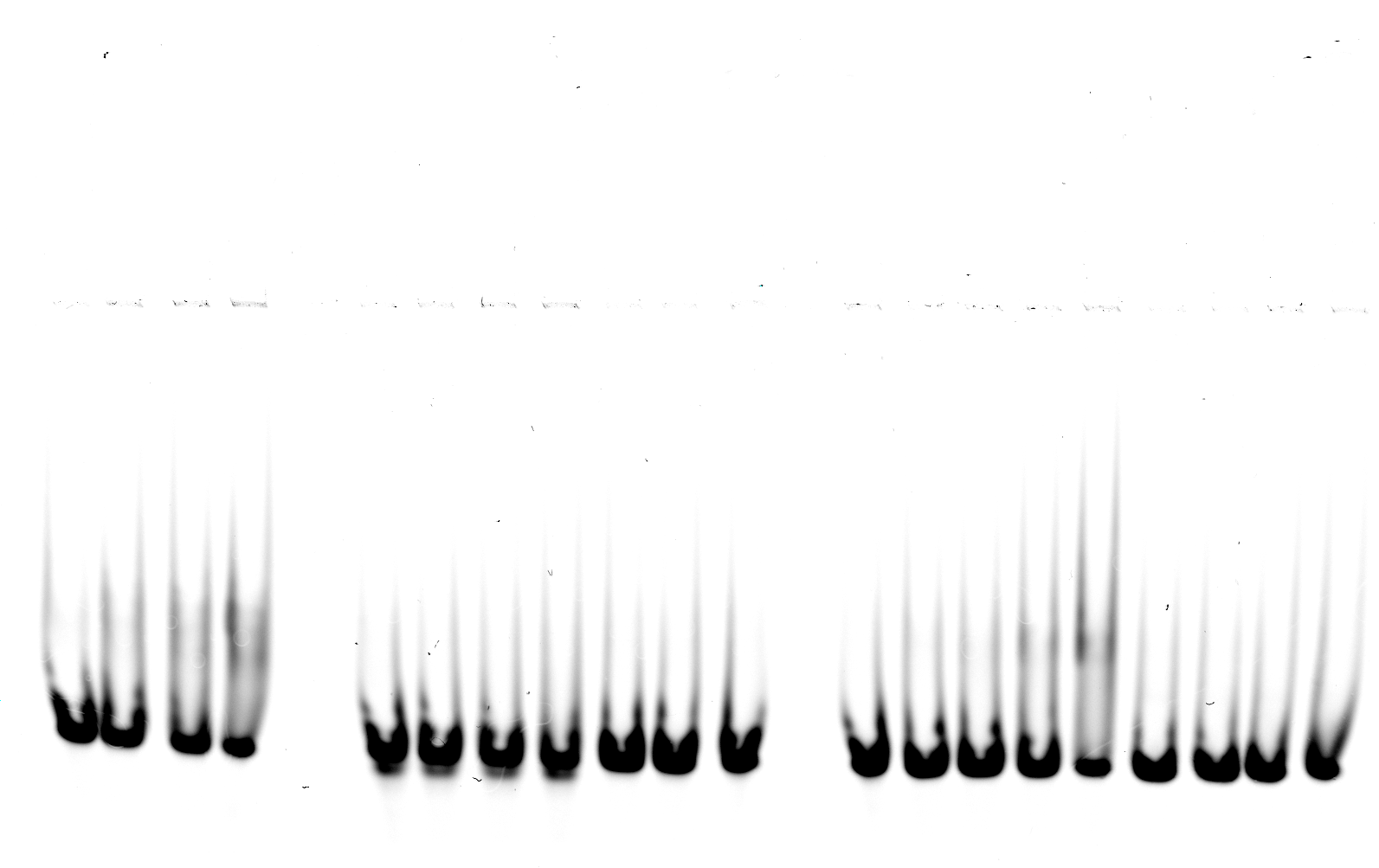

Supplement: Figure 5—figure supplement 1—source data 1. [file elife-70833-fig5-figsupp1-data1.zip › Figure5 - figure supplement 1 - source data 1/Figure 5C and Figure 5-S1B - Source data 1.tif]

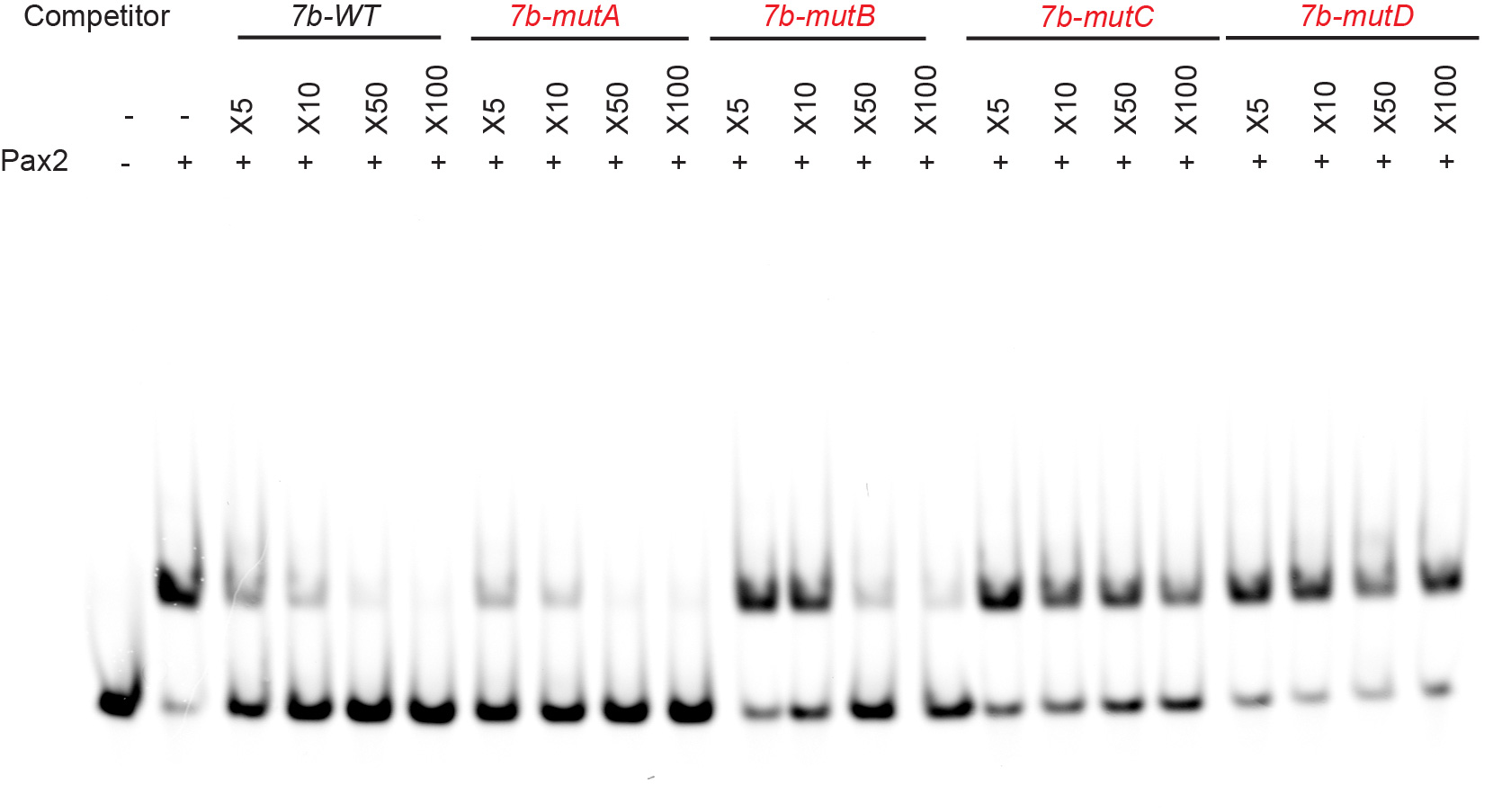

Supplement: Figure 5—figure supplement 1—source data 1. [file elife-70833-fig5-figsupp1-data1.zip › Figure5 - figure supplement 1 - source data 1/Figure 5-S1E - Source data 1_labeled.jpg]

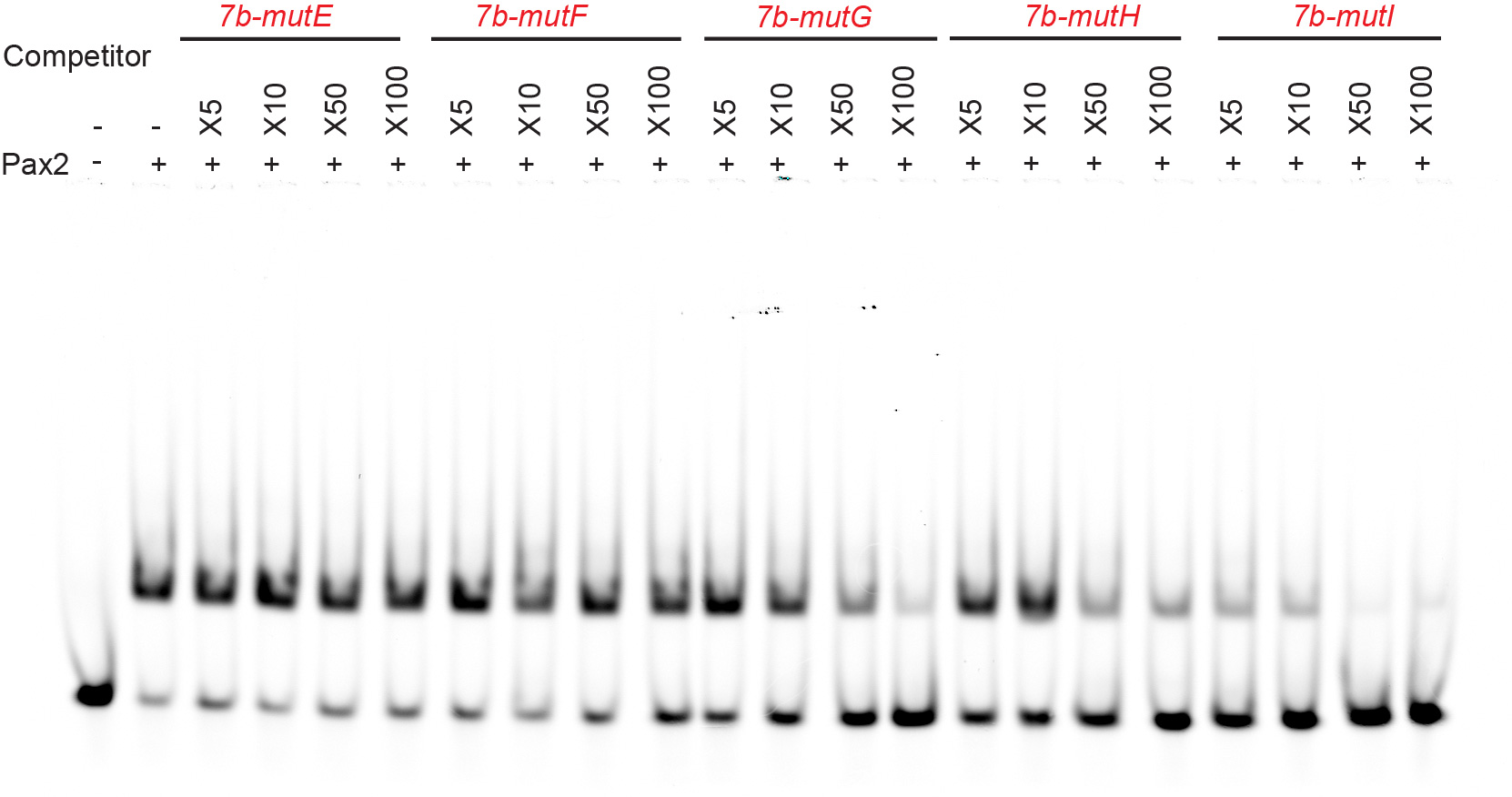

Supplement: Figure 5—figure supplement 1—source data 1. [file elife-70833-fig5-figsupp1-data1.zip › Figure5 - figure supplement 1 - source data 1/Figure 5-S1E - Source data 2_labeled.jpg]

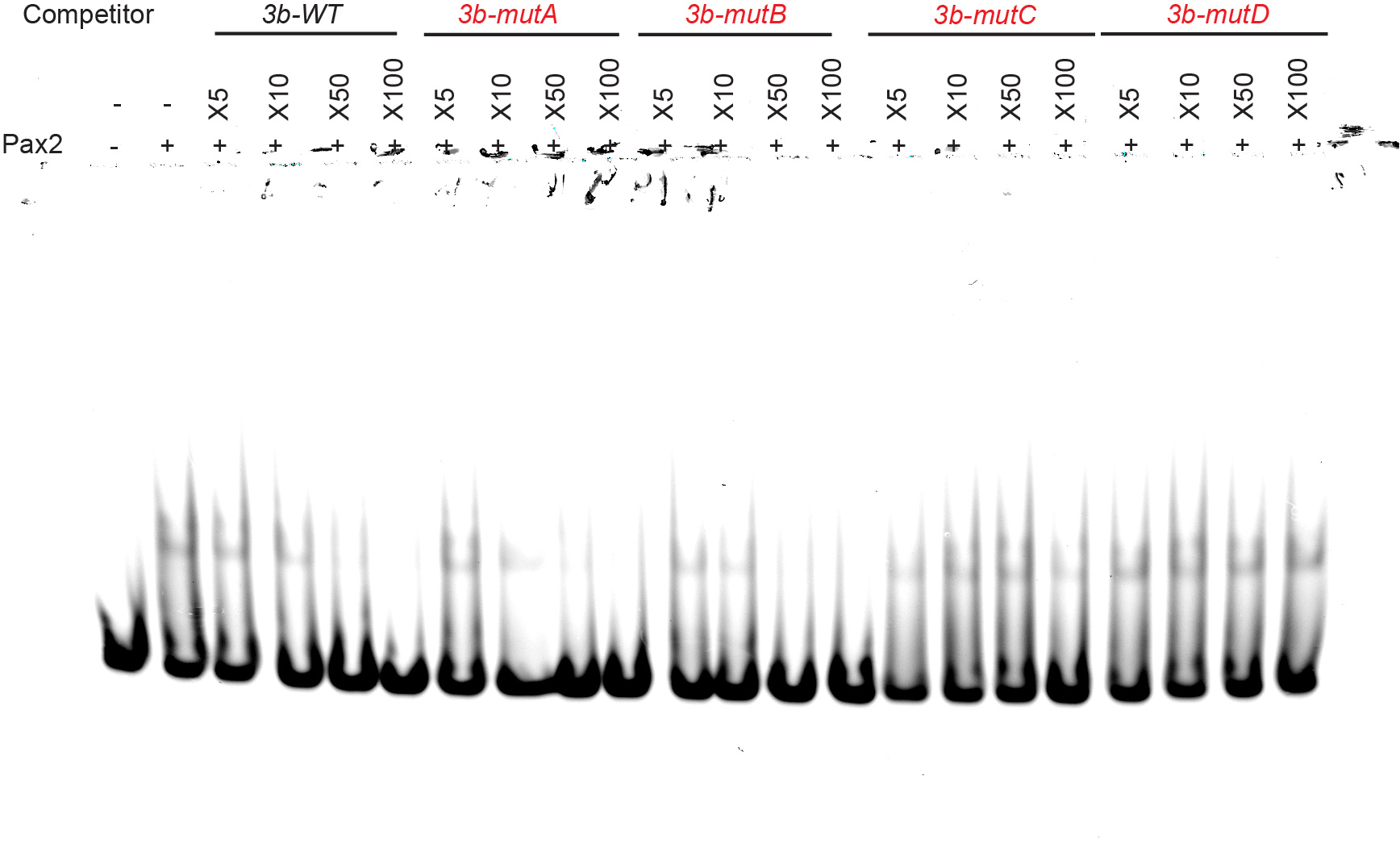

Supplement: Figure 5—figure supplement 1—source data 1. [file elife-70833-fig5-figsupp1-data1.zip › Figure5 - figure supplement 1 - source data 1/Figure 5-S1D - Source data 1_labeled.jpg]

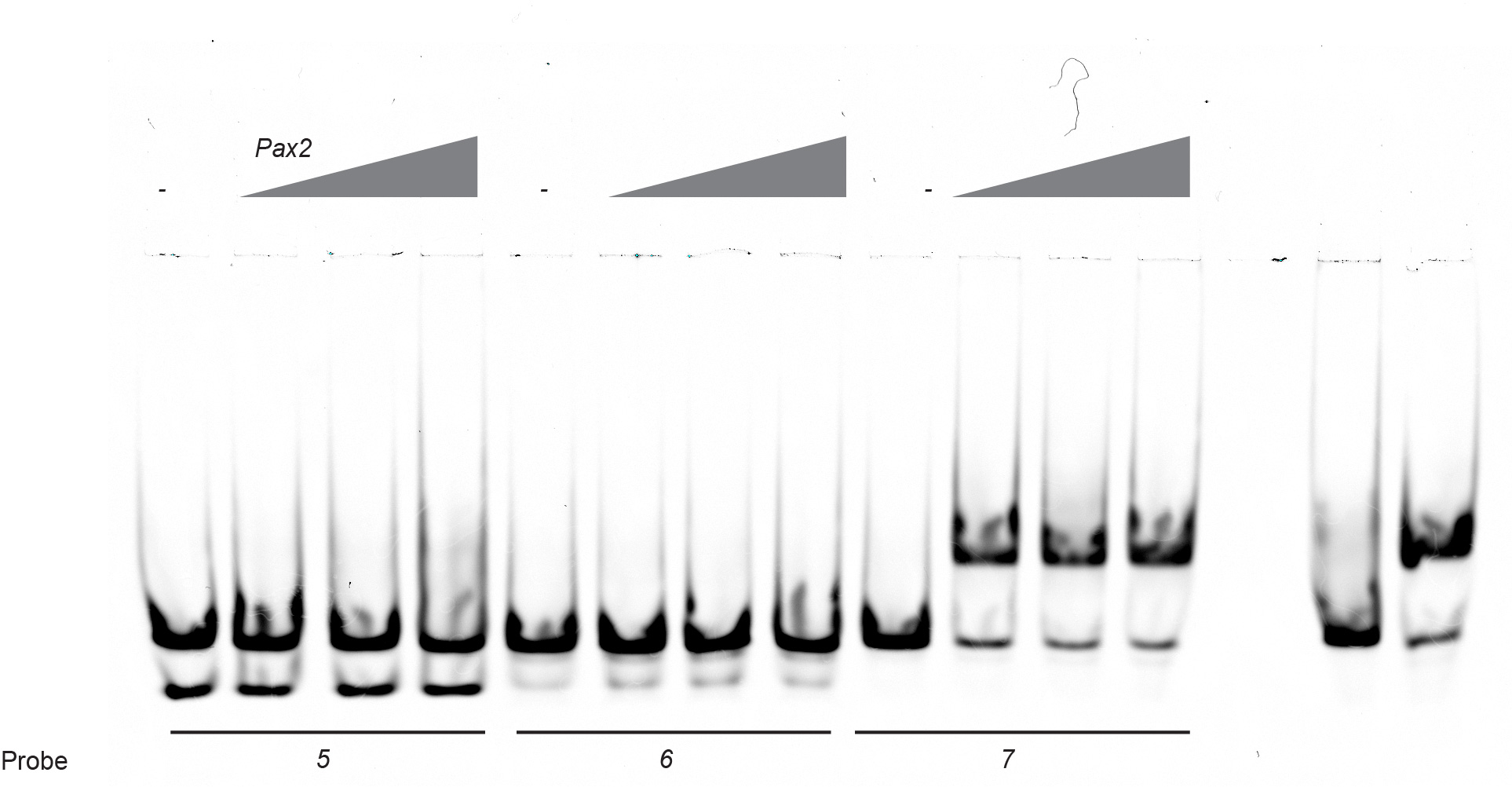

Supplement: Figure 5—figure supplement 1—source data 1. [file elife-70833-fig5-figsupp1-data1.zip › Figure5 - figure supplement 1 - source data 1/Figure 5C and Figure 5-S1B - Source data 2_labeled.jpg]

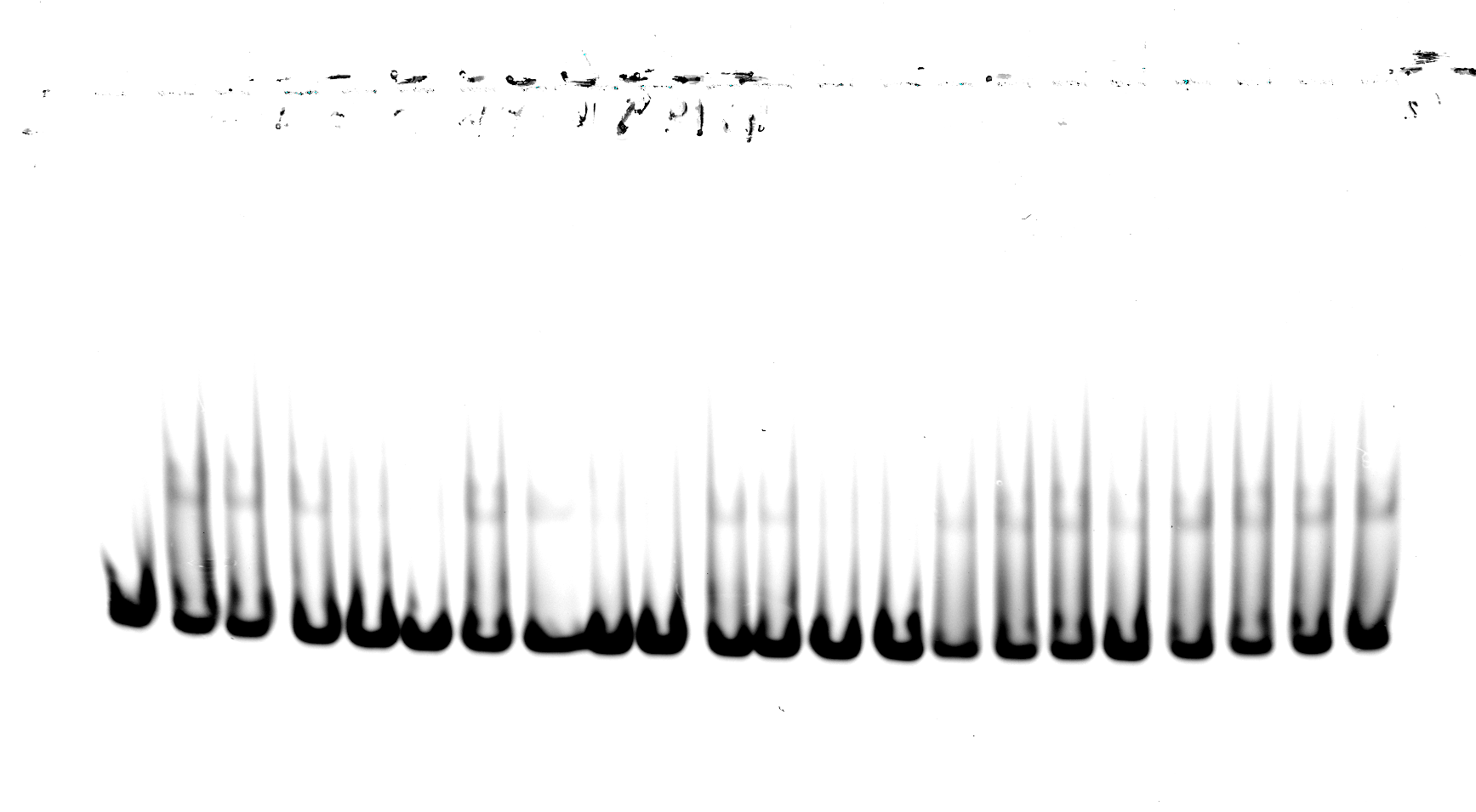

Supplement: Figure 5—figure supplement 1—source data 1. [file elife-70833-fig5-figsupp1-data1.zip › Figure5 - figure supplement 1 - source data 1/Figure 5-S1D - Source data 1.tif]

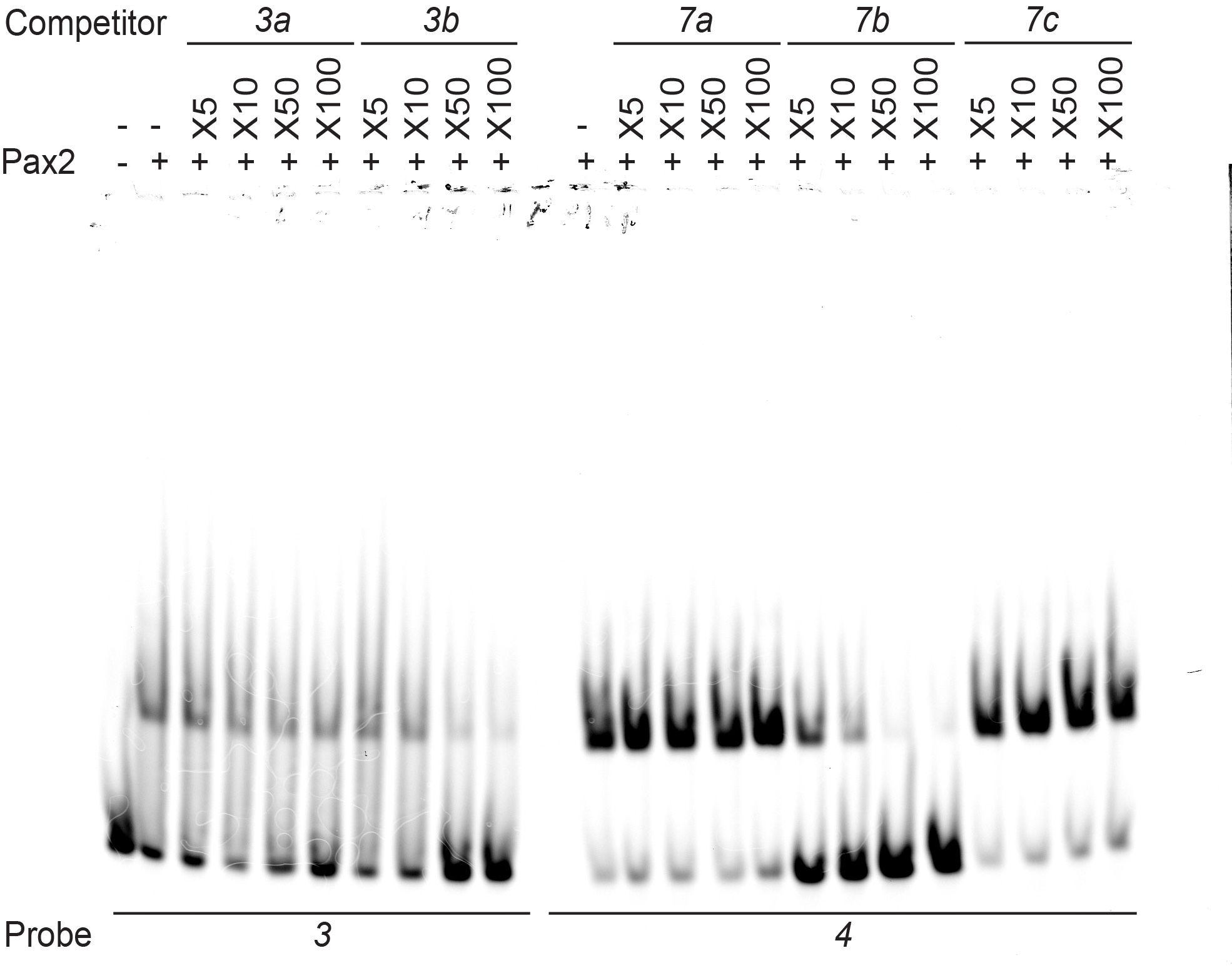

Supplement: Figure 5—figure supplement 1—source data 1. [file elife-70833-fig5-figsupp1-data1.zip › Figure5 - figure supplement 1 - source data 1/Figure 5-S1C - Source data 1_labeled.jpg]

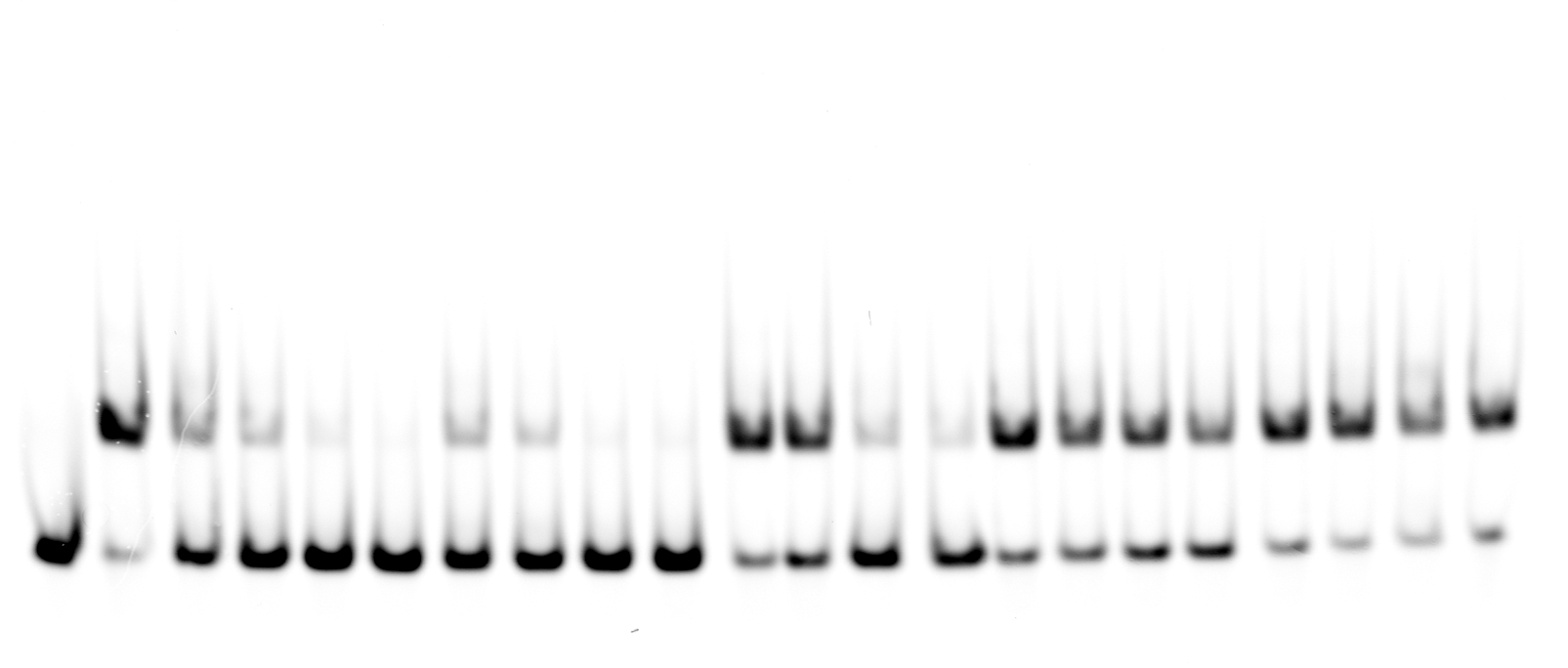

Supplement: Figure 5—figure supplement 1—source data 1. [file elife-70833-fig5-figsupp1-data1.zip › Figure5 - figure supplement 1 - source data 1/Figure 5-S1E - Source data 1.tif]

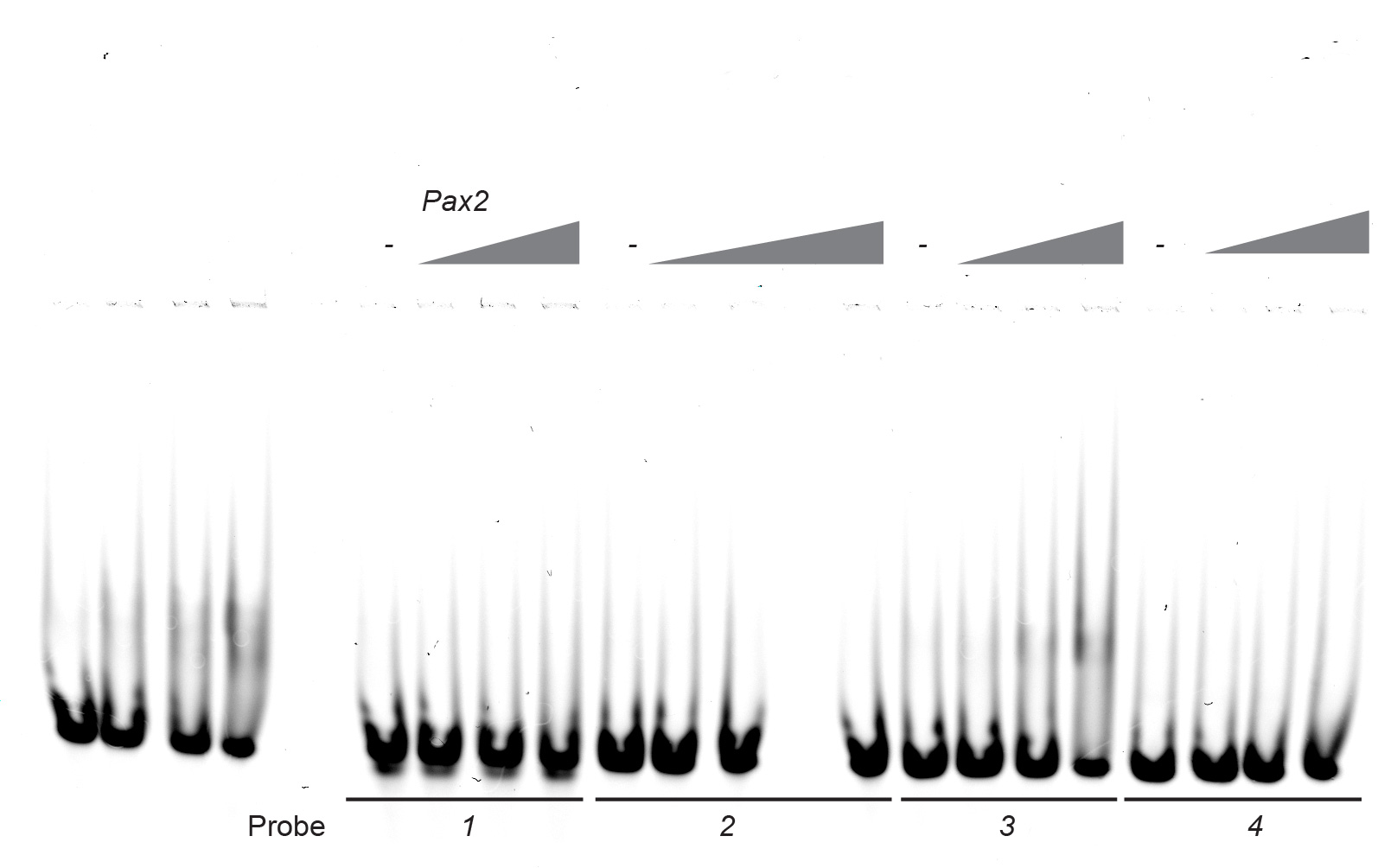

Supplement: Figure 5—figure supplement 1—source data 1. [file elife-70833-fig5-figsupp1-data1.zip › Figure5 - figure supplement 1 - source data 1/Figure 5C and Figure 5-S1B - Source data 1_labeled.jpg]

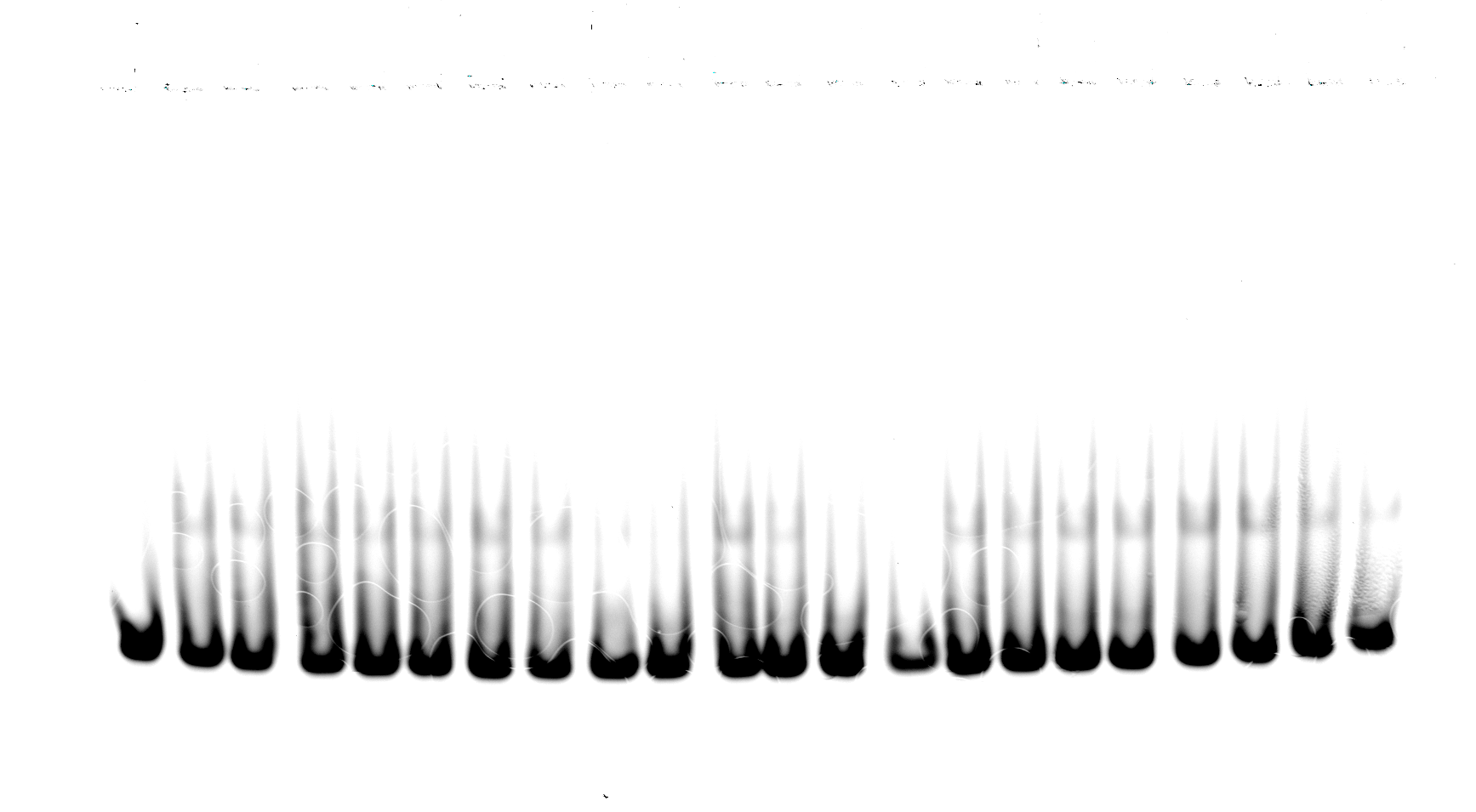

Supplement: Figure 5—figure supplement 1—source data 1. [file elife-70833-fig5-figsupp1-data1.zip › Figure5 - figure supplement 1 - source data 1/Figure 5-S1D - Source data 2.tif]

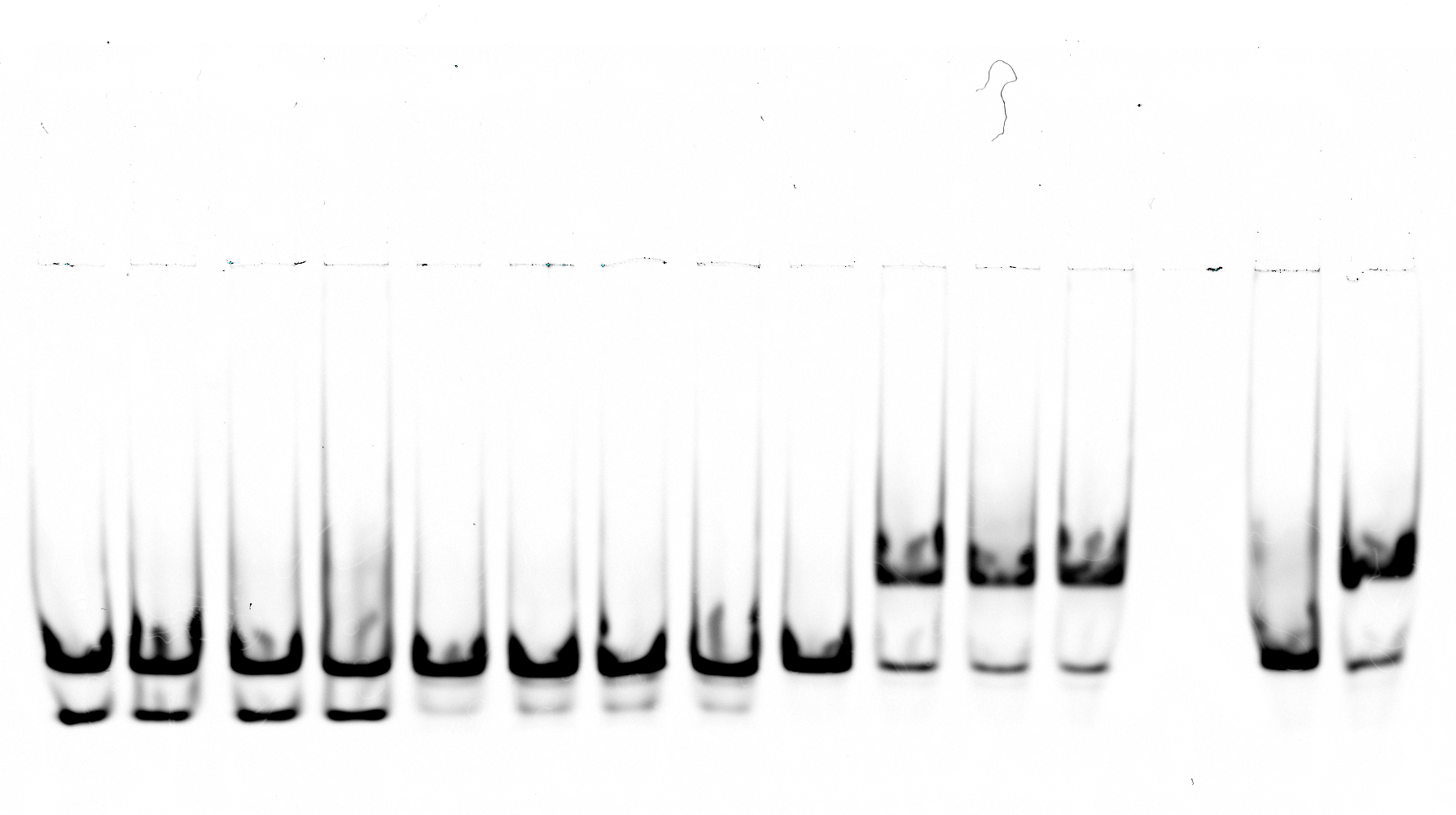

Supplement: Figure 5—figure supplement 1—source data 1. [file elife-70833-fig5-figsupp1-data1.zip › Figure5 - figure supplement 1 - source data 1/Figure 5C and Figure5-S1B - Source data 2.tif]

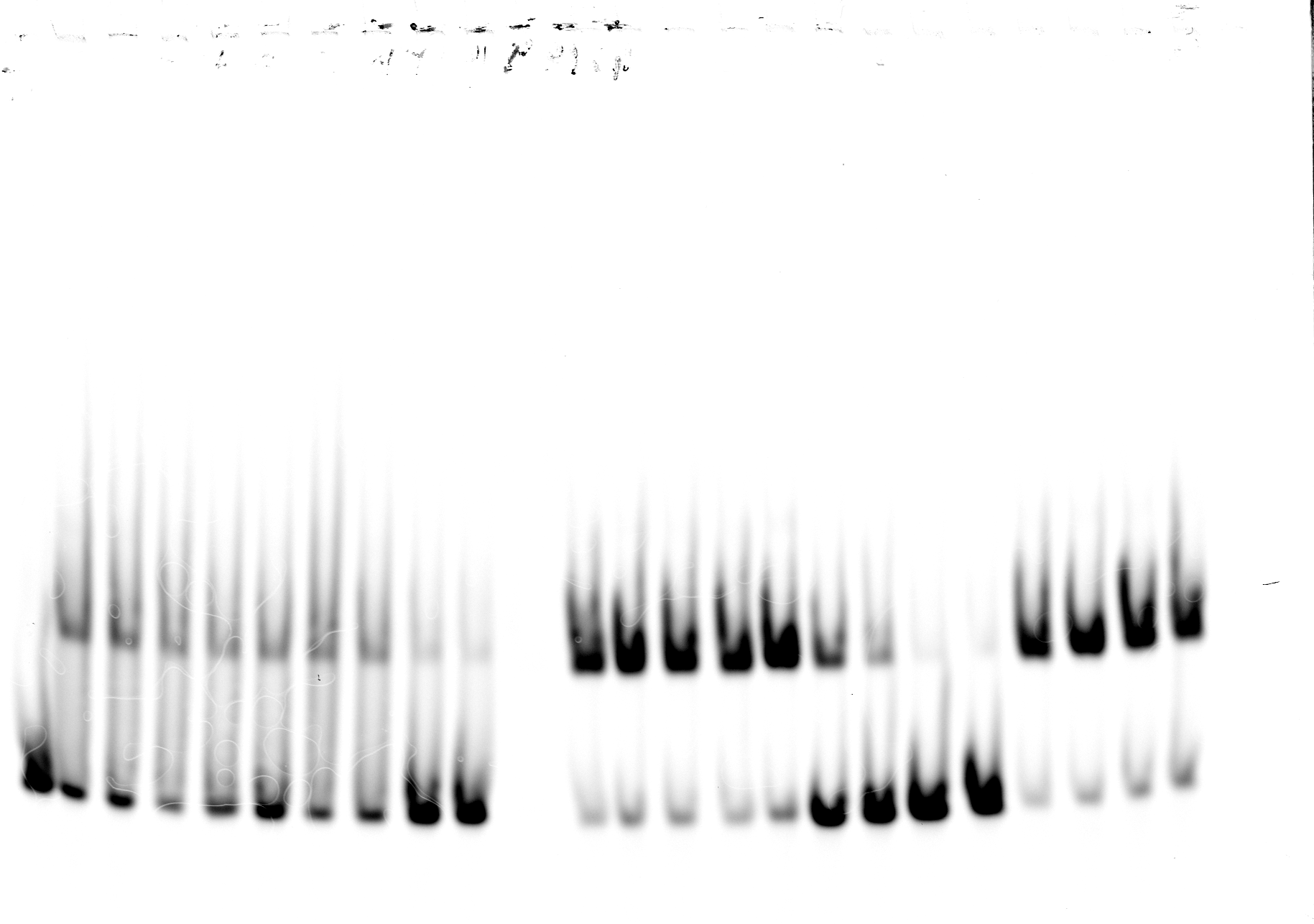

Supplement: Figure 5—figure supplement 1—source data 1. [file elife-70833-fig5-figsupp1-data1.zip › Figure5 - figure supplement 1 - source data 1/Figure 5-S1C - Source data 1.tif]

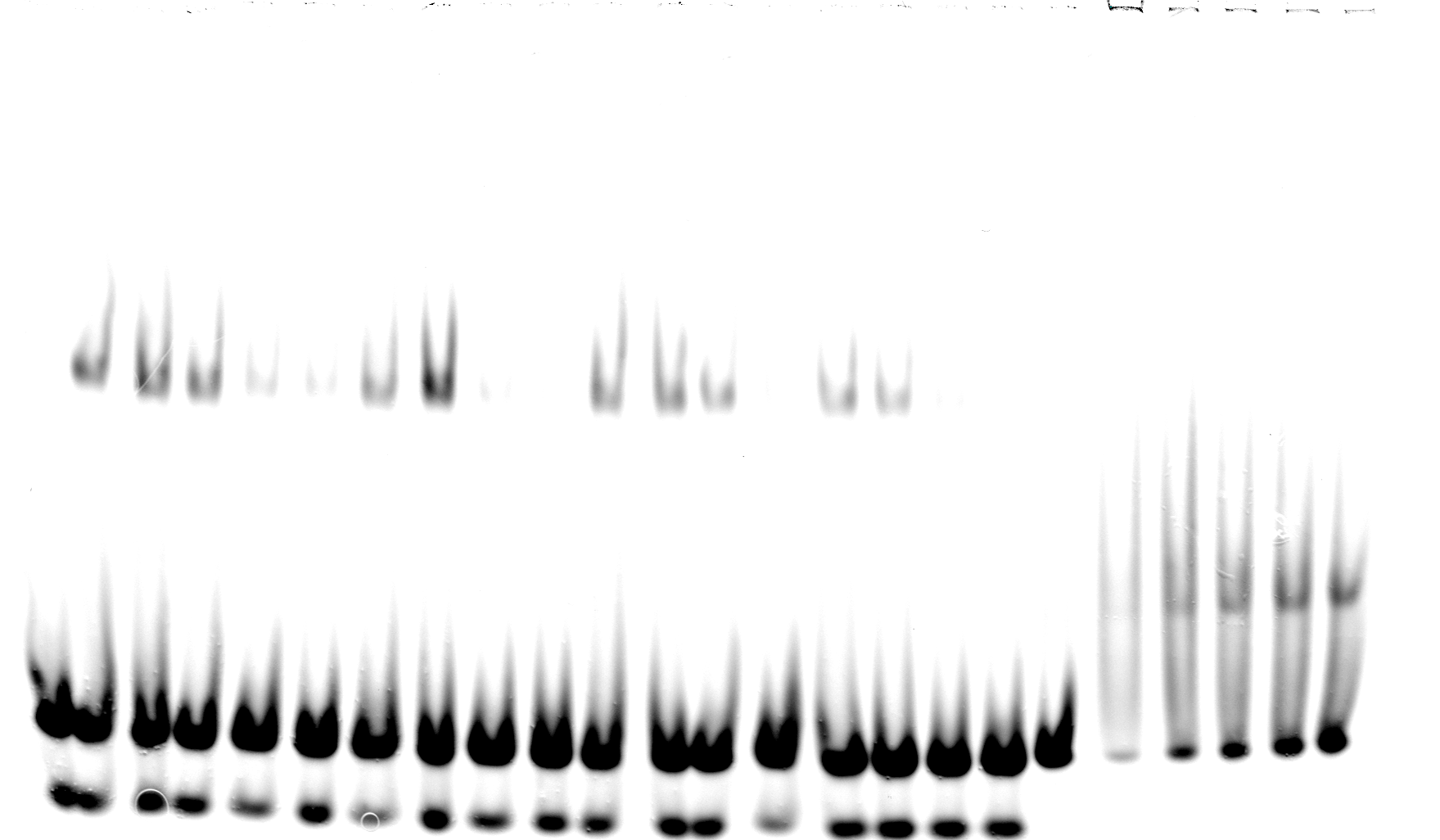

Supplement: Figure 5—figure supplement 2—source data 1. [file elife-70833-fig5-figsupp2-data1.zip › Figure5 - figure supplement 2 - source data 1/Figure 5-S2E - Source data 4.tif]

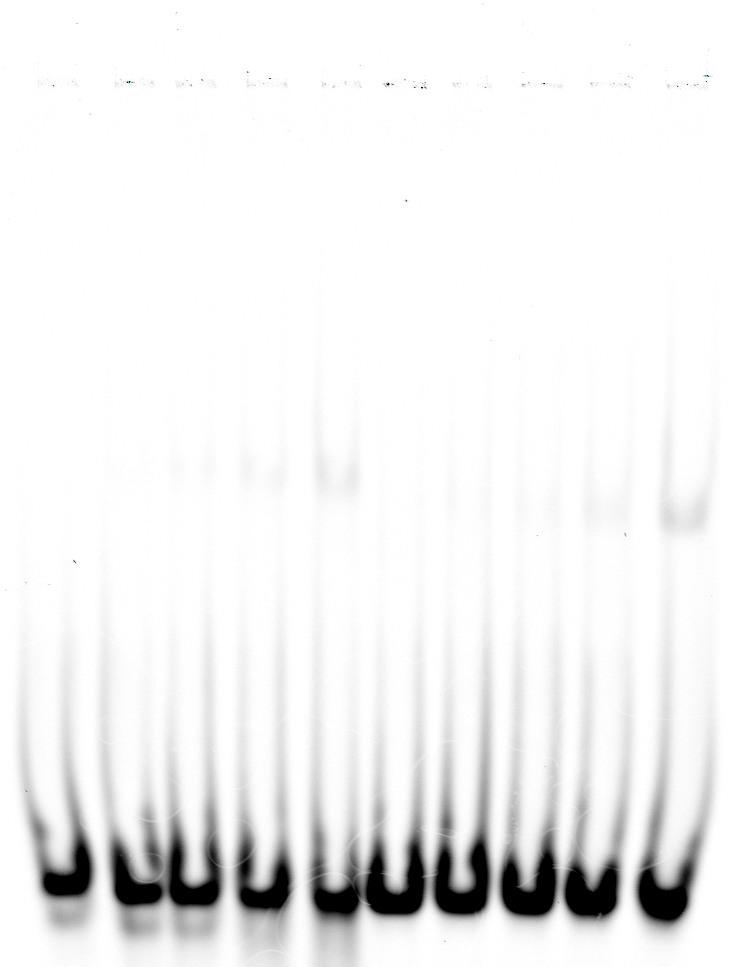

Supplement: Figure 5—figure supplement 2—source data 1. [file elife-70833-fig5-figsupp2-data1.zip › Figure5 - figure supplement 2 - source data 1/Figure 5-S2B - Source data 1.tif]

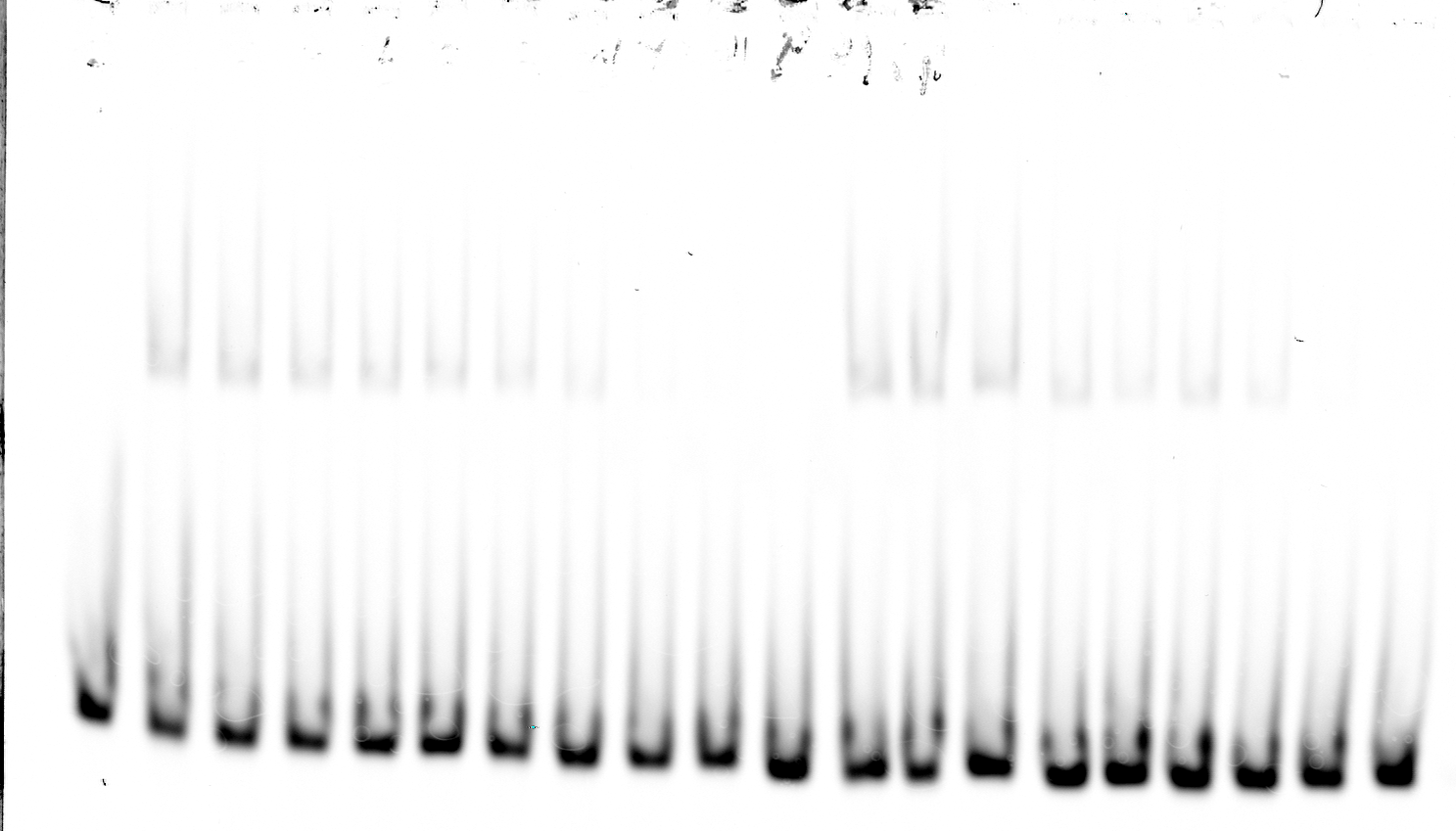

Supplement: Figure 5—figure supplement 2—source data 1. [file elife-70833-fig5-figsupp2-data1.zip › Figure5 - figure supplement 2 - source data 1/Figure 5-S2C - Source data 1.tif]

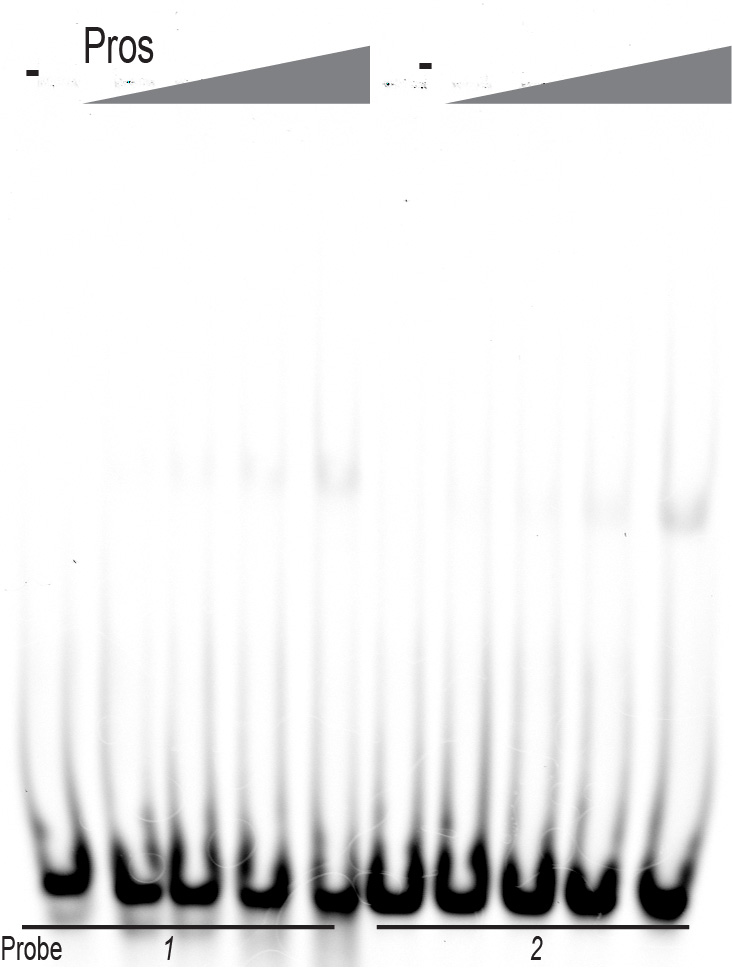

Supplement: Figure 5—figure supplement 2—source data 1. [file elife-70833-fig5-figsupp2-data1.zip › Figure5 - figure supplement 2 - source data 1/Figure 5-S2 - Source data 1_labeled.jpg]

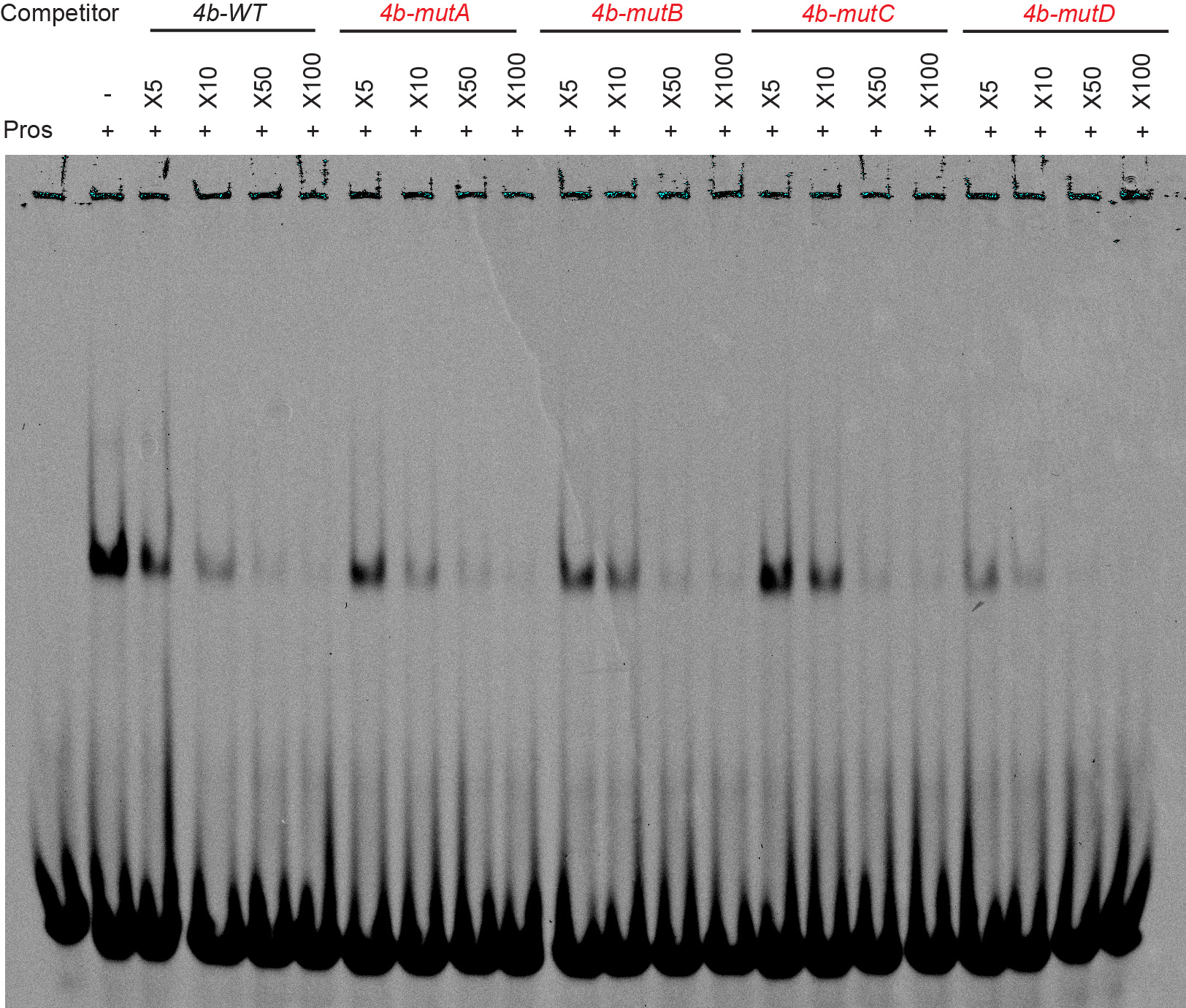

Supplement: Figure 5—figure supplement 2—source data 1. [file elife-70833-fig5-figsupp2-data1.zip › Figure5 - figure supplement 2 - source data 1/Figure 5-S2E - Source data 1_labeled.jpg]

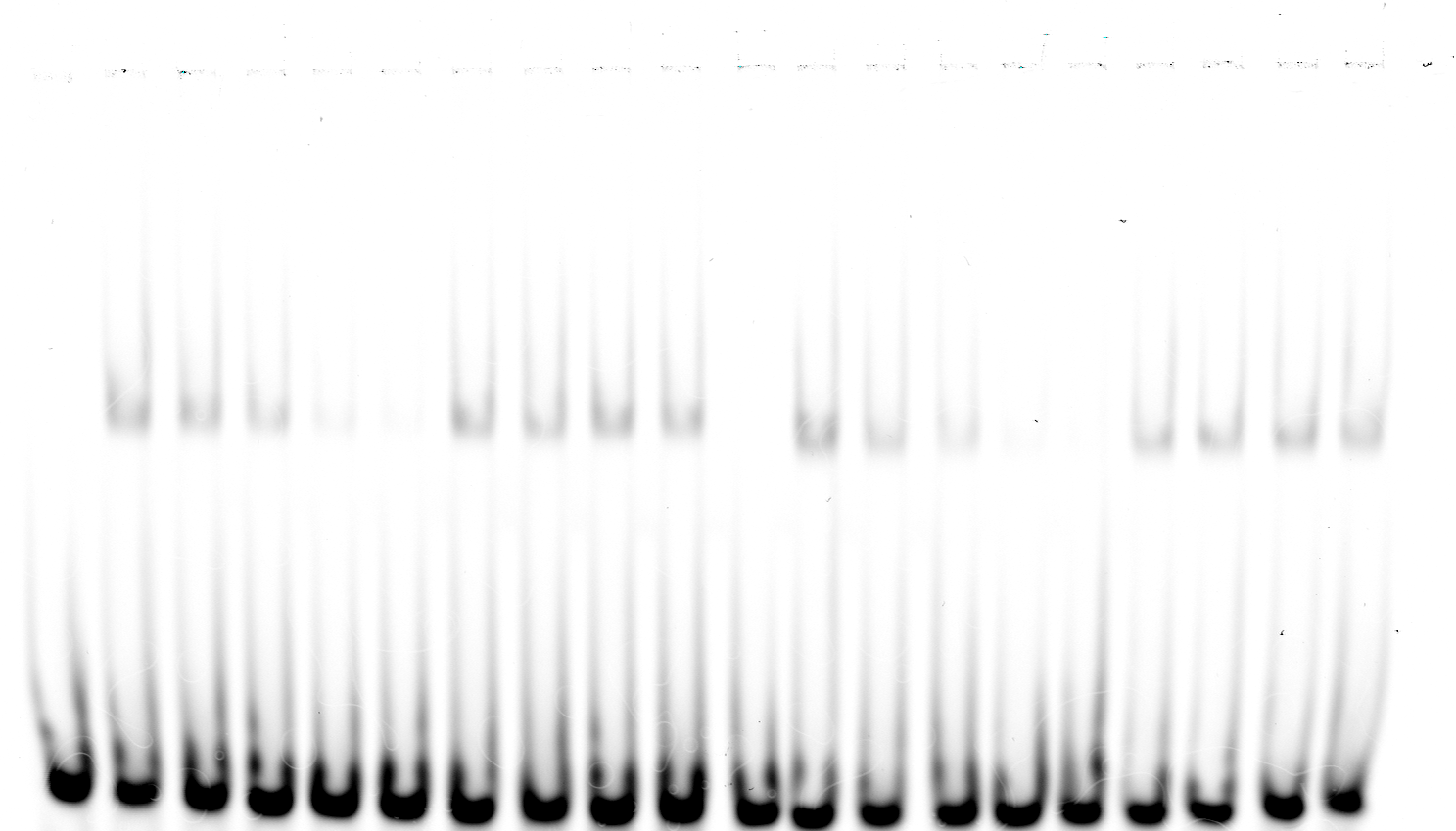

Supplement: Figure 5—figure supplement 2—source data 1. [file elife-70833-fig5-figsupp2-data1.zip › Figure5 - figure supplement 2 - source data 1/Figure 5-S2C - Source data 2.tif]

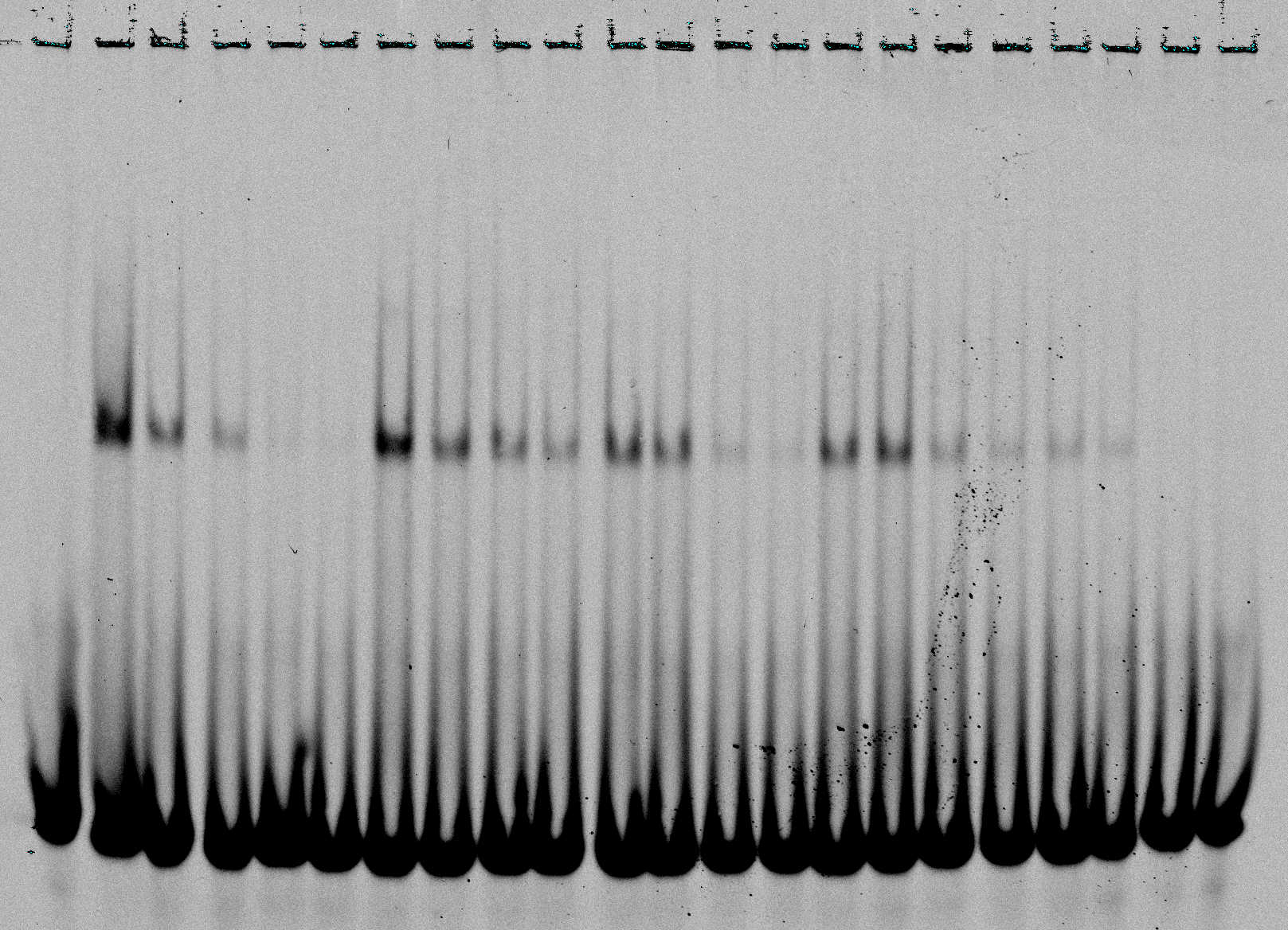

Supplement: Figure 5—figure supplement 2—source data 1. [file elife-70833-fig5-figsupp2-data1.zip › Figure5 - figure supplement 2 - source data 1/Figure 5-S2E - Source data 2.tif]

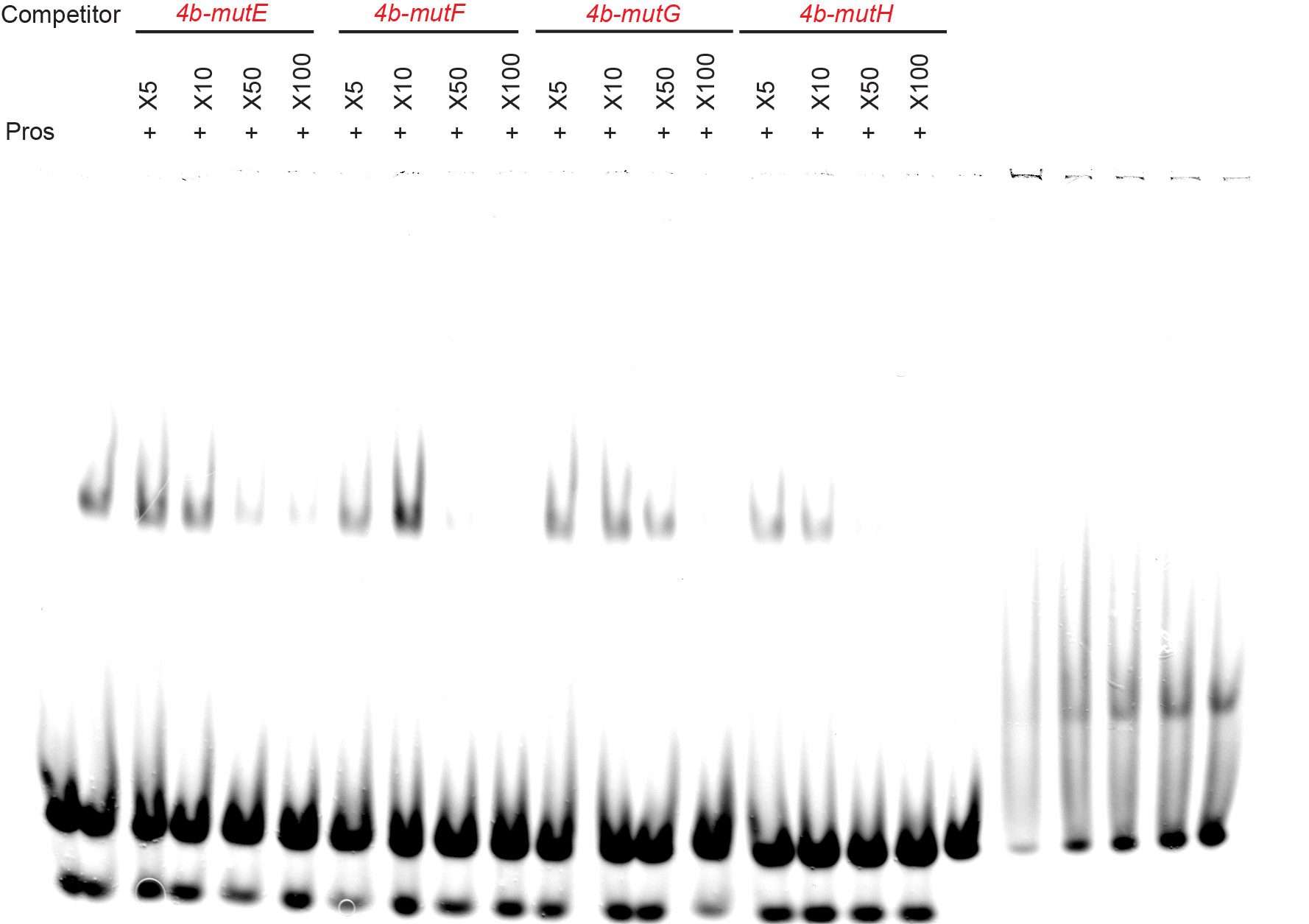

Supplement: Figure 5—figure supplement 2—source data 1. [file elife-70833-fig5-figsupp2-data1.zip › Figure5 - figure supplement 2 - source data 1/Figure 5-S2E - Source data 4_labeled.jpg]

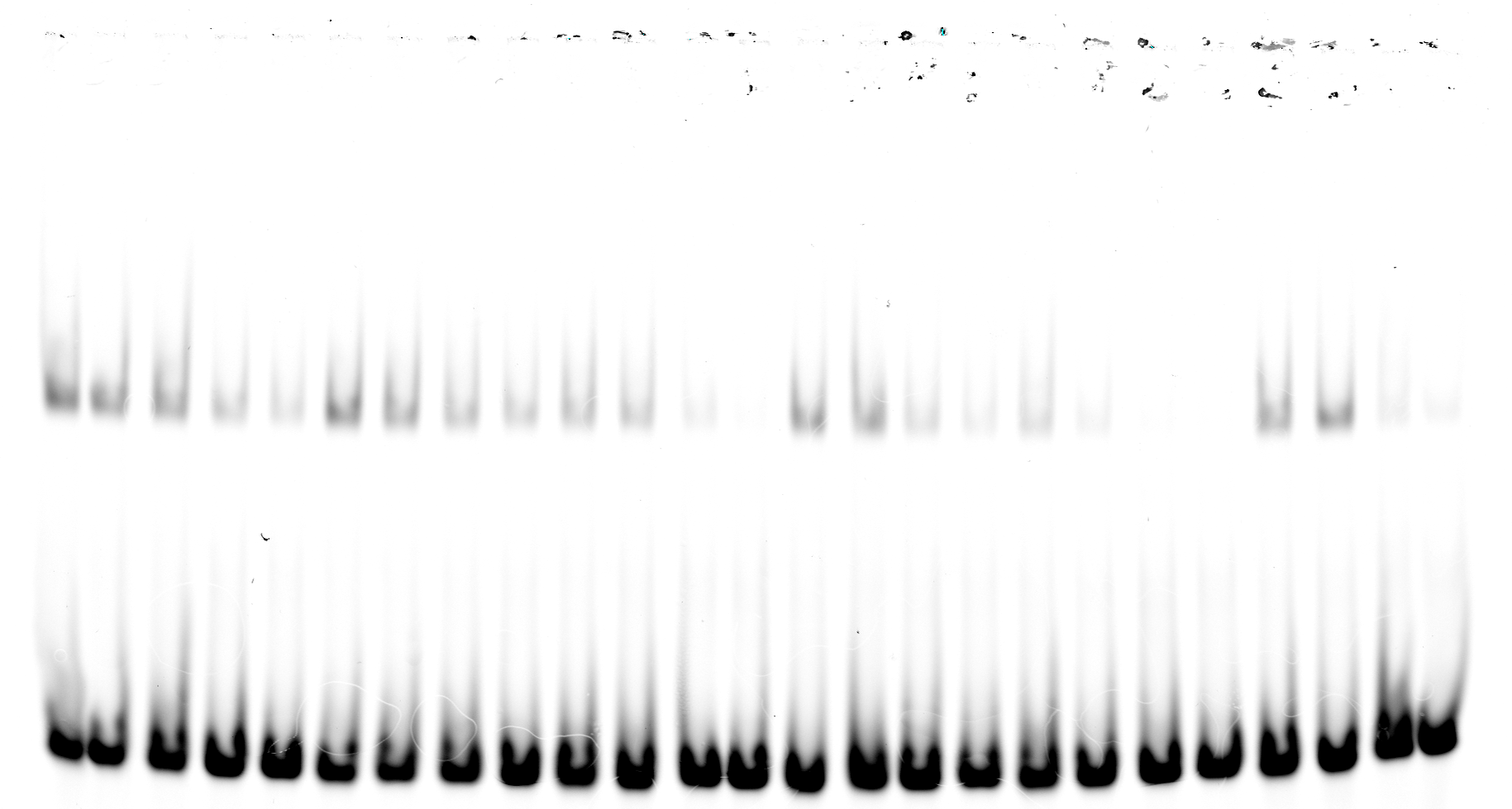

Supplement: Figure 5—figure supplement 2—source data 1. [file elife-70833-fig5-figsupp2-data1.zip › Figure5 - figure supplement 2 - source data 1/Figure 5-S2D - Source data 2.tif]

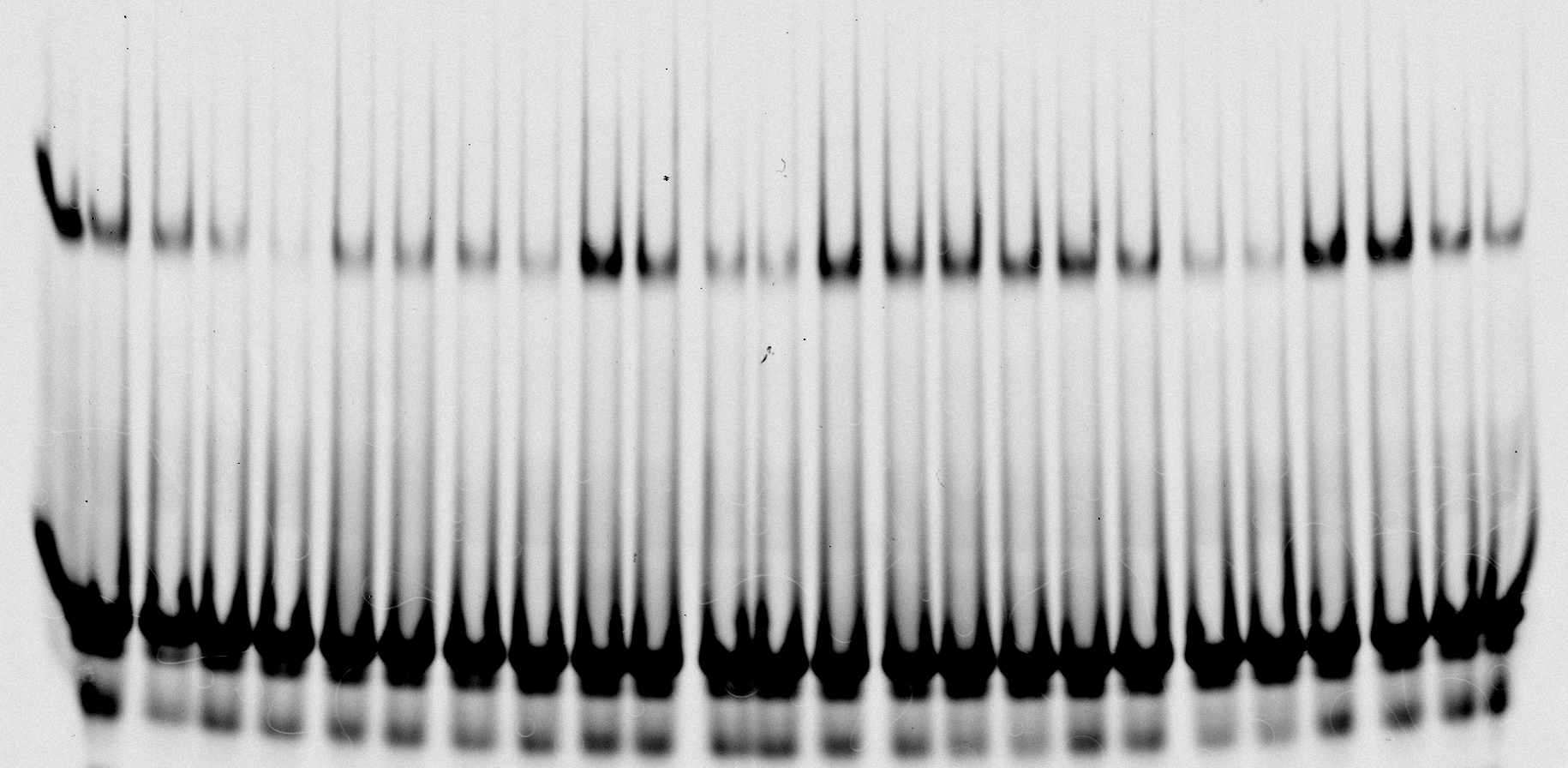

Supplement: Figure 5—figure supplement 2—source data 1. [file elife-70833-fig5-figsupp2-data1.zip › Figure5 - figure supplement 2 - source data 1/Figure 5-S2F - Source data 1.tif]

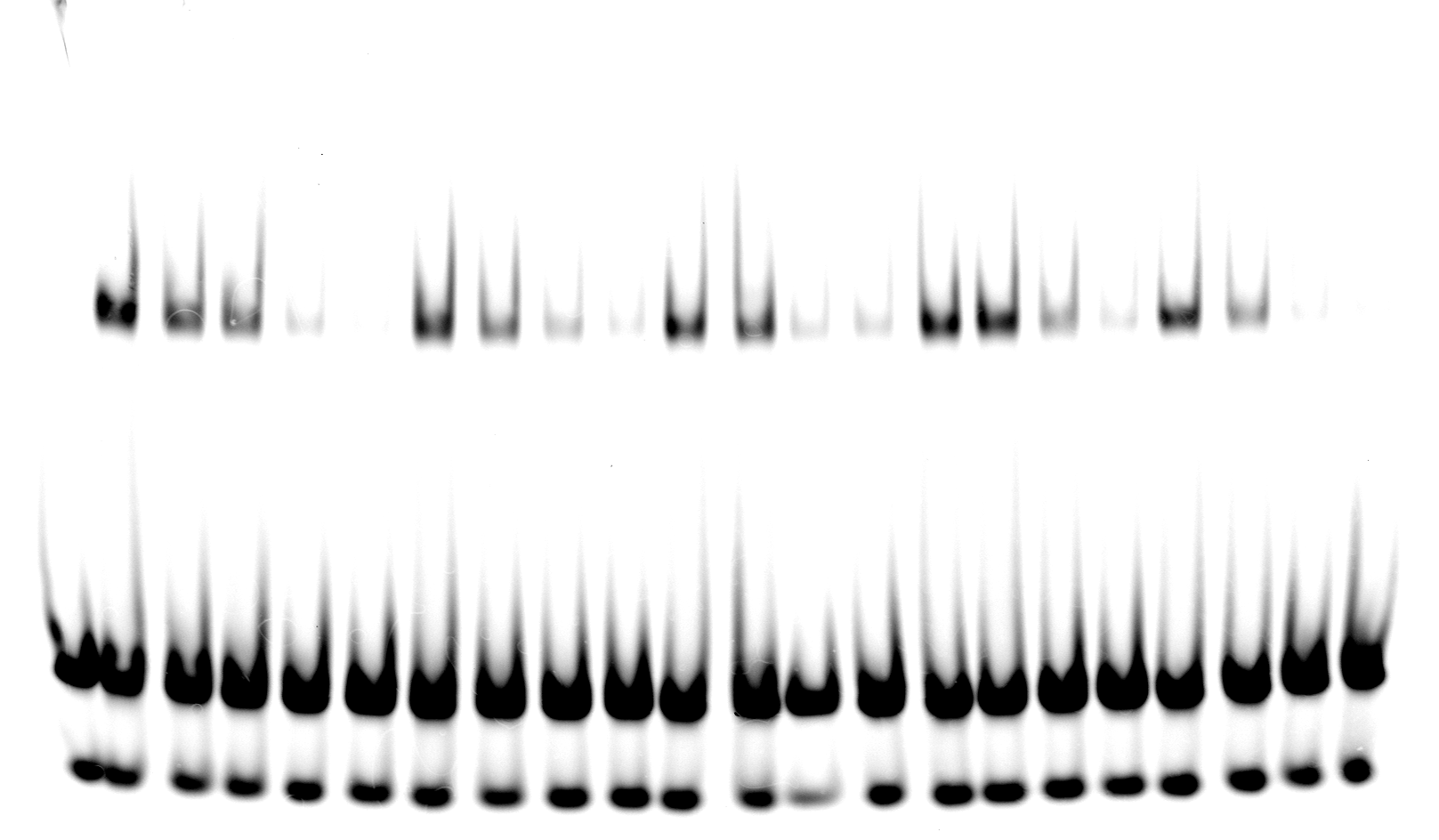

Supplement: Figure 5—figure supplement 2—source data 1. [file elife-70833-fig5-figsupp2-data1.zip › Figure5 - figure supplement 2 - source data 1/Figure 5-S2E - Source data 3.tif]

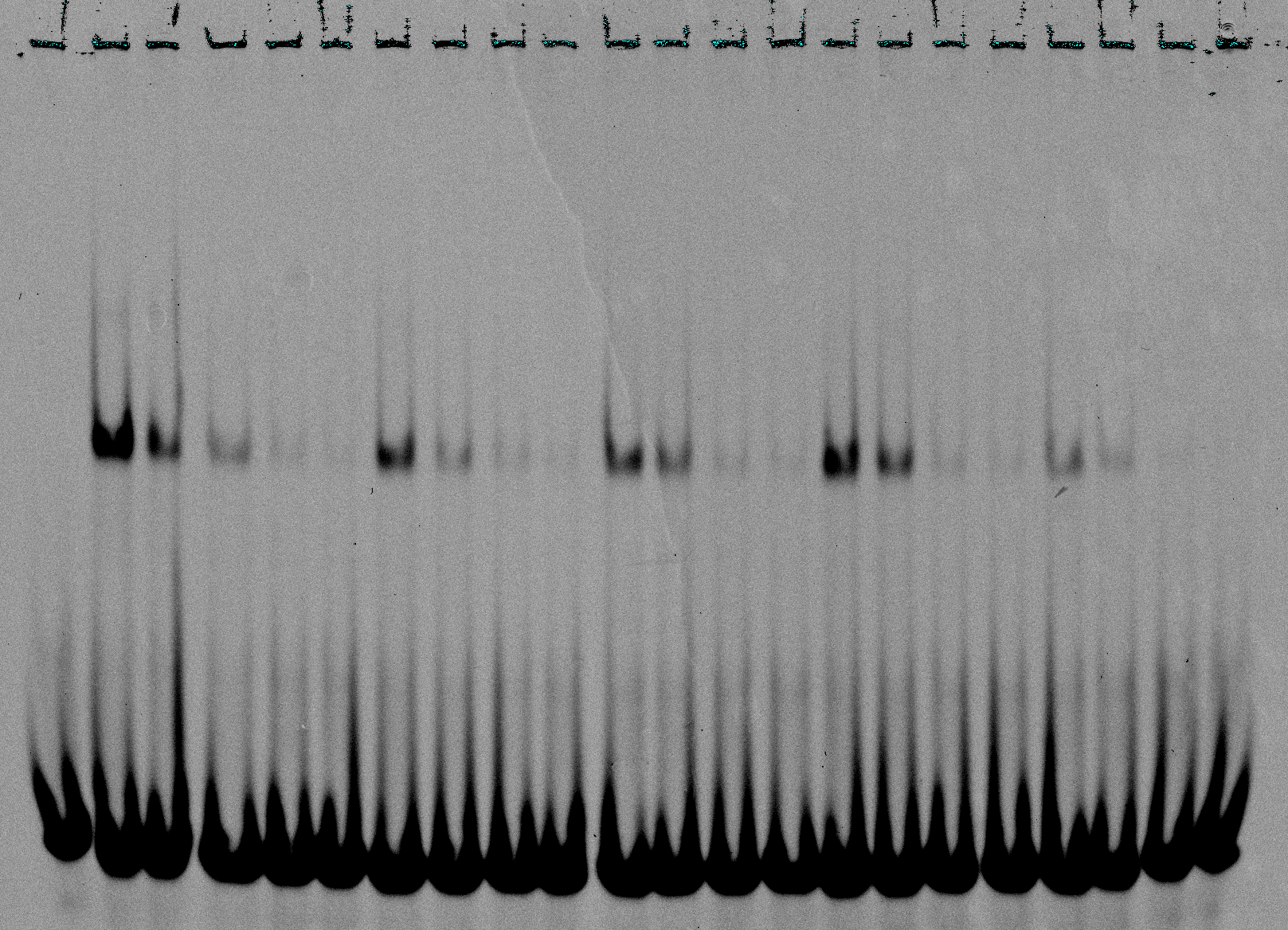

Supplement: Figure 5—figure supplement 2—source data 1. [file elife-70833-fig5-figsupp2-data1.zip › Figure5 - figure supplement 2 - source data 1/Figure 5-S2E - Source data 1.tif]

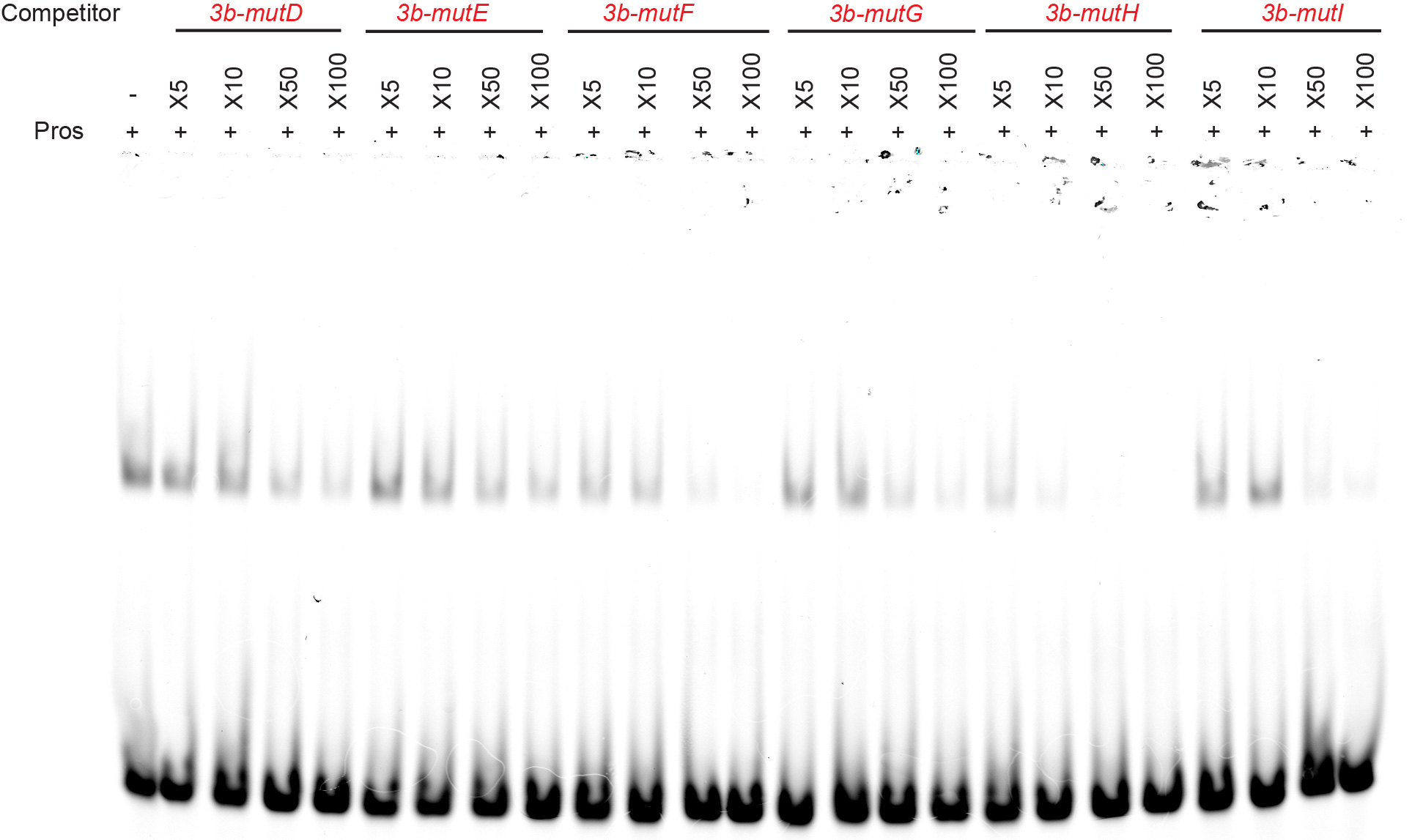

Supplement: Figure 5—figure supplement 2—source data 1. [file elife-70833-fig5-figsupp2-data1.zip › Figure5 - figure supplement 2 - source data 1/Figure 5-S2D - Source data 2_labeled.jpg]

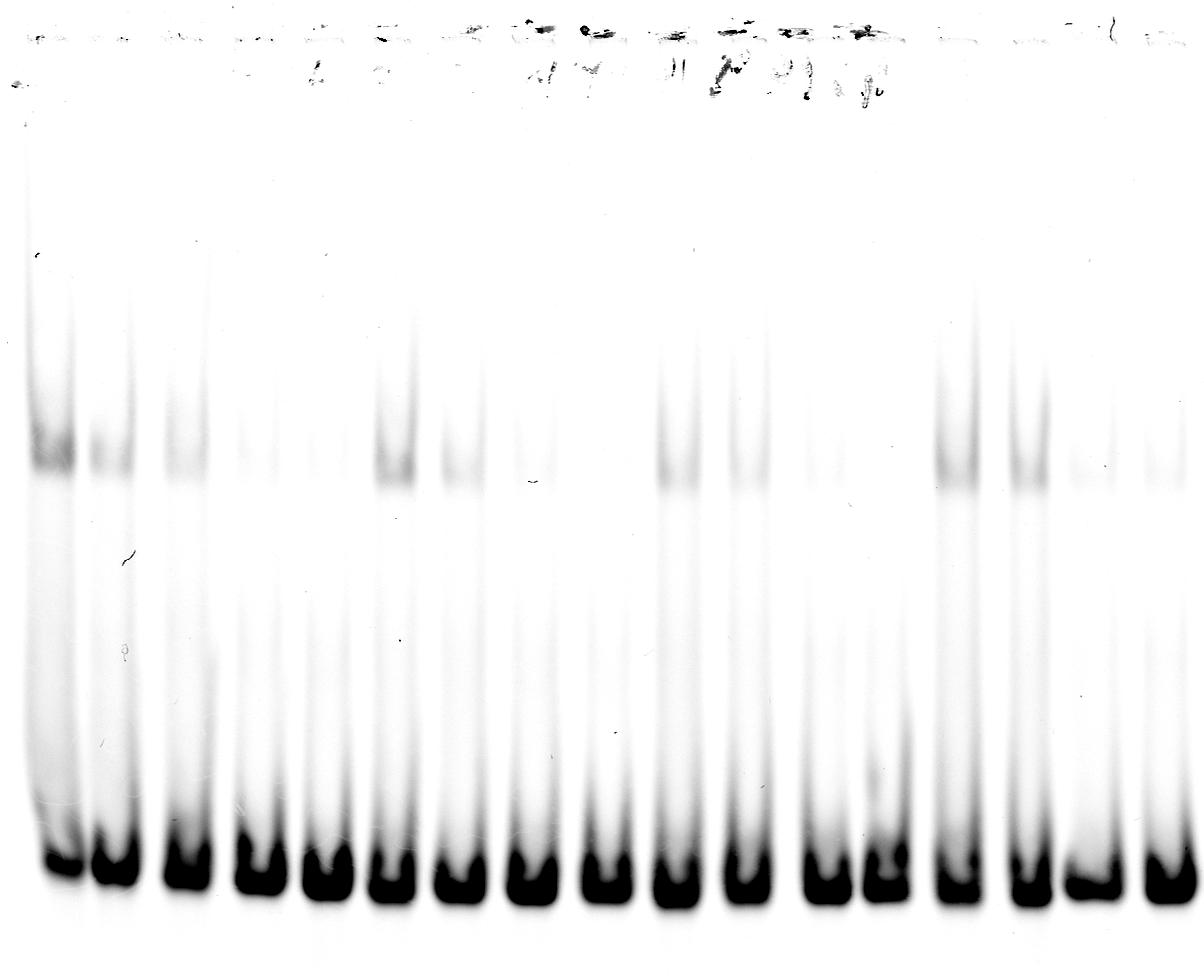

Supplement: Figure 5—figure supplement 2—source data 1. [file elife-70833-fig5-figsupp2-data1.zip › Figure5 - figure supplement 2 - source data 1/Figure 5-S2D - Source data 1.tif]

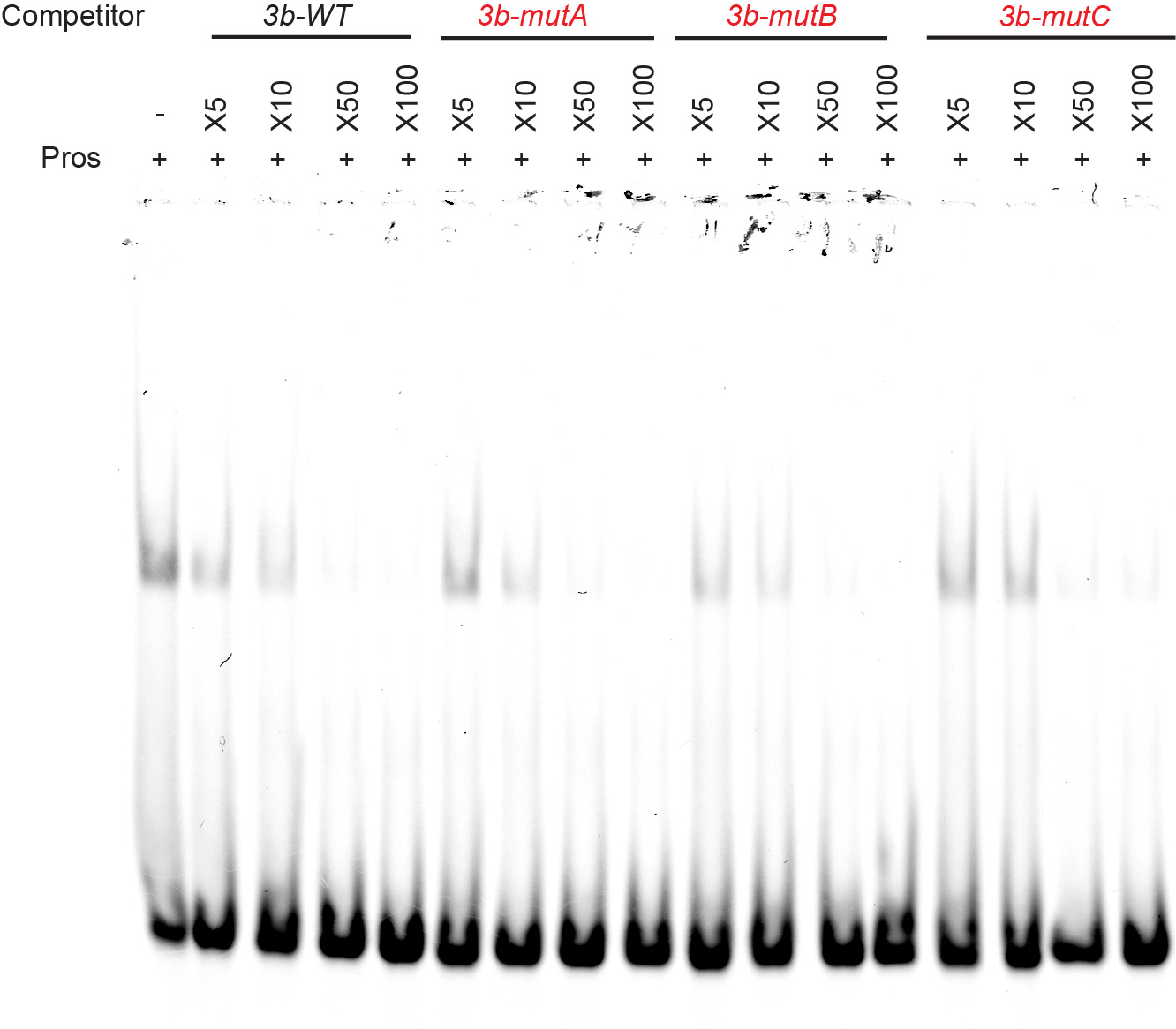

Supplement: Figure 5—figure supplement 2—source data 1. [file elife-70833-fig5-figsupp2-data1.zip › Figure5 - figure supplement 2 - source data 1/Figure 5-S2D - Source data 1_labeled.jpg]

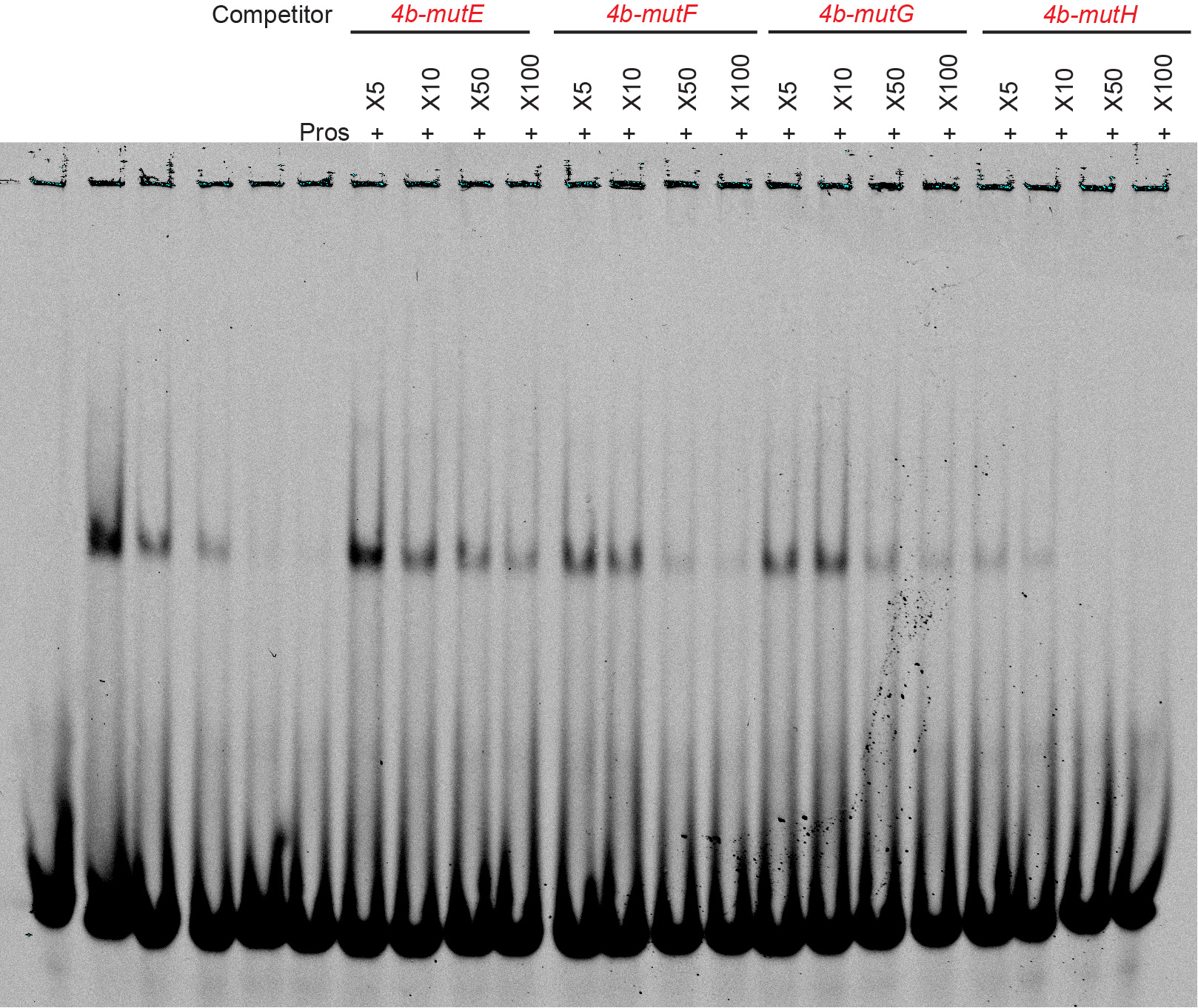

Supplement: Figure 5—figure supplement 2—source data 1. [file elife-70833-fig5-figsupp2-data1.zip › Figure5 - figure supplement 2 - source data 1/Figure 5-S2E - Source data 2_labeled.jpg]

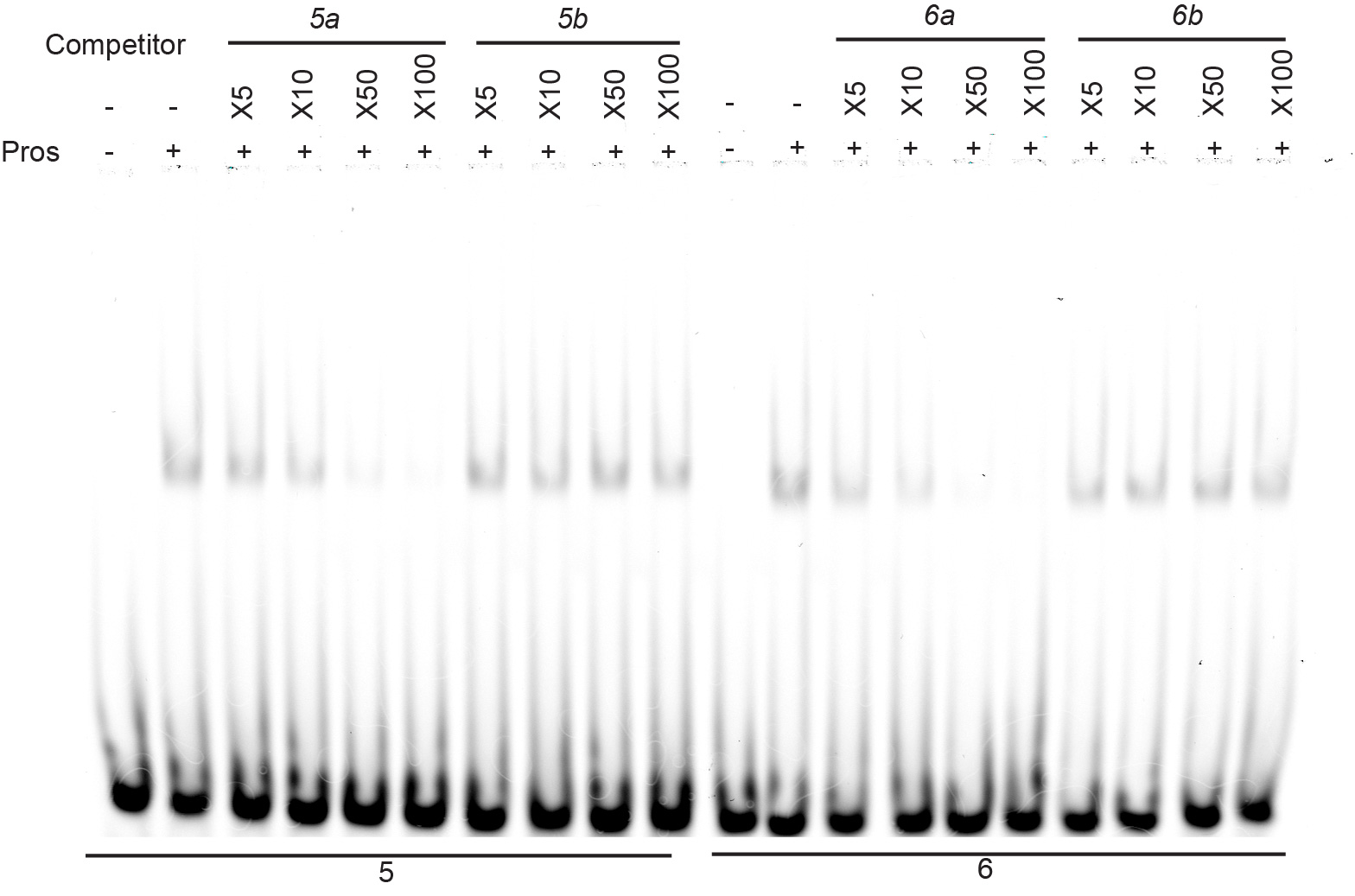

Supplement: Figure 5—figure supplement 2—source data 1. [file elife-70833-fig5-figsupp2-data1.zip › Figure5 - figure supplement 2 - source data 1/Figure 5-S2C - Source data 2_labeled.jpg]

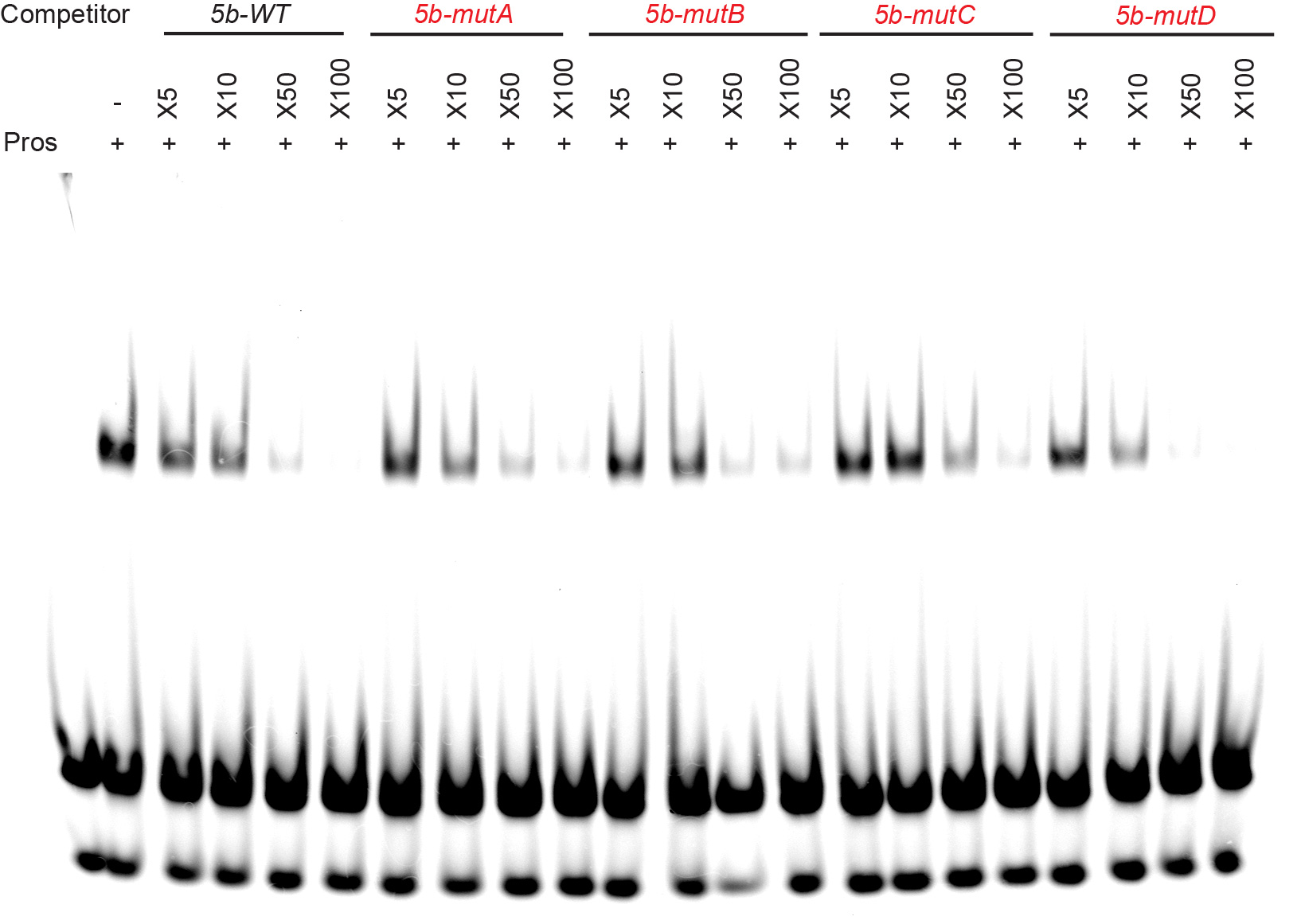

Supplement: Figure 5—figure supplement 2—source data 1. [file elife-70833-fig5-figsupp2-data1.zip › Figure5 - figure supplement 2 - source data 1/Figure 5-S2E - Source data 3_labeled.jpg]

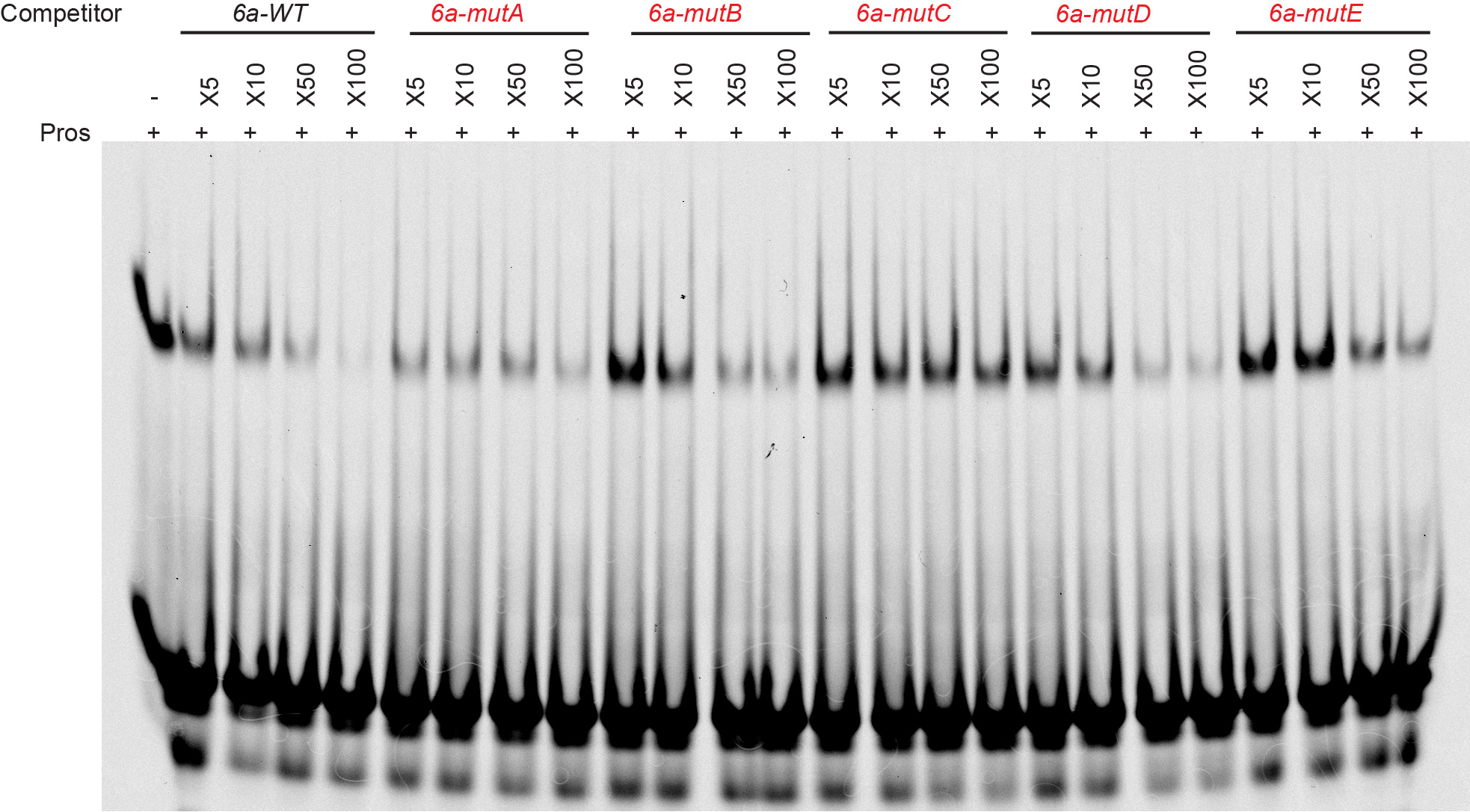

Supplement: Figure 5—figure supplement 2—source data 1. [file elife-70833-fig5-figsupp2-data1.zip › Figure5 - figure supplement 2 - source data 1/Figure 5-S2F - Source data 1_labeled.jpg]

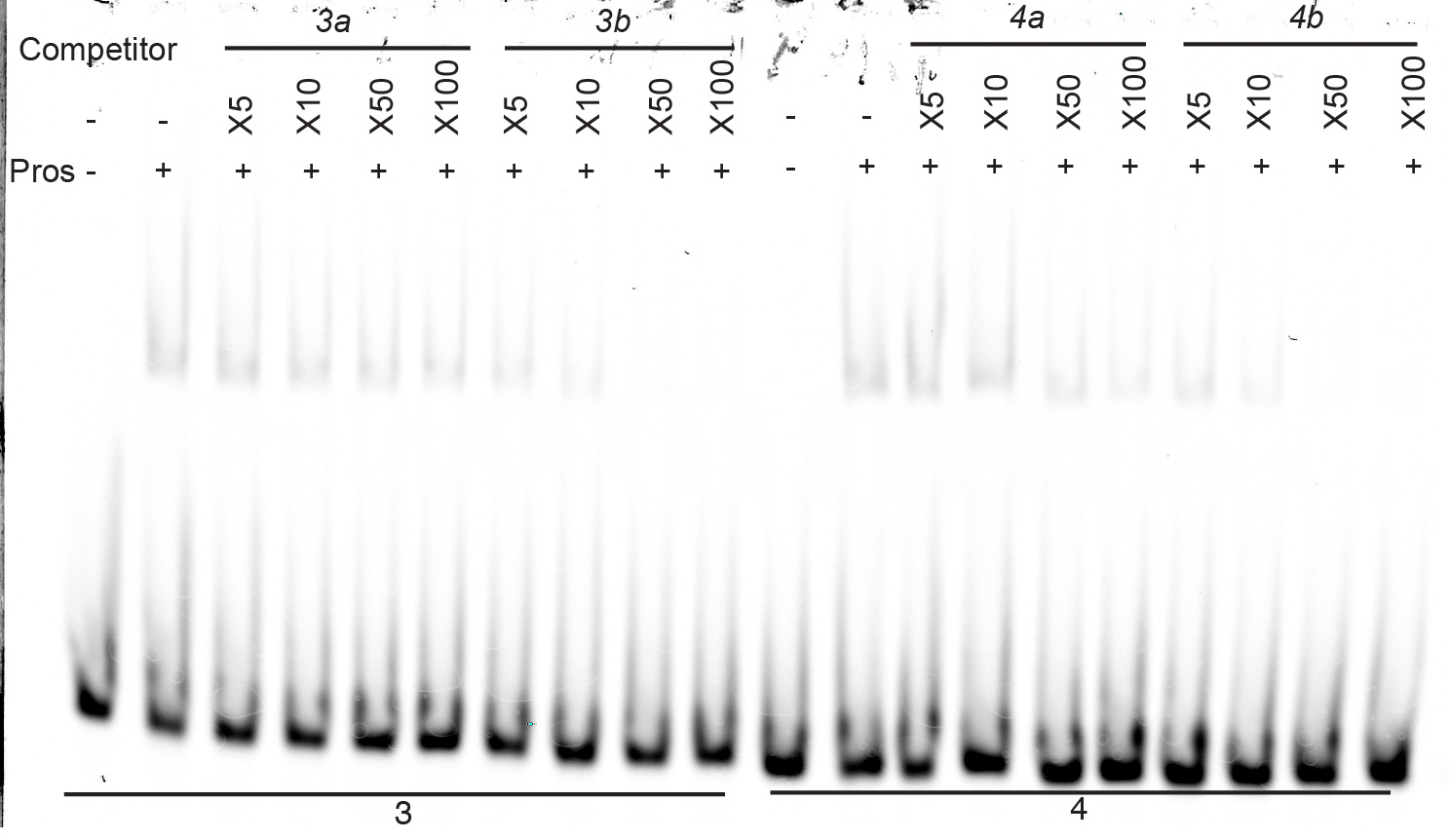

Supplement: Figure 5—figure supplement 2—source data 1. [file elife-70833-fig5-figsupp2-data1.zip › Figure5 - figure supplement 2 - source data 1/Figure 5-S2C - Source data 1_labeled.jpg]
